# Fluorescent probes towards selective cathepsin B detection and visualization in cancer cells and patient samples†
†Electronic supplementary information (ESI) available. See DOI: 10.1039/c9sc00997c


**DOI:** 10.1039/c9sc00997c

**Published:** 2019-07-31

**Authors:** Marcin Poreba, Katarzyna Groborz, Matej Vizovisek, Marco Maruggi, Dusan Turk, Boris Turk, Garth Powis, Marcin Drag, Guy S. Salvesen

**Affiliations:** a Sanford Burnham Prebys Medical Discovery Institute , 10901 North Torrey Pines Road , La Jolla , CA 92037 , USA . Email: marcin.poreba@pwr.edu.pl ; Email: marcin.drag@pwr.edu.pl ; Email: gsalvesen@sbpdiscovery.org; b Department of Bioorganic Chemistry , Faculty of Chemistry , Wroclaw University of Technology , Wyb. Wyspianskiego 27 , 50-370 Wroclaw , Poland; c Department of Biochemistry and Molecular and Structural Biology , Jožef Stefan Institute , SI-1000 Ljubljana , Slovenia; d Faculty of Chemistry and Chemical Technology , University of Ljubljana , SI-1000 Ljubljana , Slovenia

## Abstract

Human cysteine cathepsins constitute an 11-membered family of proteases responsible for degradation of proteins in cellular endosomal–lysosomal compartments as such, they play important roles in antigen processing, cellular stress signaling, autophagy, and senescence. Moreover, for many years these enzymes were also linked to tumor growth, invasion, angiogenesis and metastasis when upregulated. Individual biological roles of each cathepsin are difficult to establish, because of their redundancy and similar substrate specificities. Selective chemical tools that enable imaging of individual cathepsin activities in living cells, tumors, and the tumor microenvironment may provide a better insight into their functions. In this work, we used HyCoSuL technology to profile the substrate specificity of human cathepsin B. The use of unnatural amino acids in the substrate library enabled us to uncover the broad cathepsin B preferences that we utilized to design highly-selective substrates and fluorescent activity-based probes (ABPs). We further demonstrated that Cy5-labeled MP-CB-2 probe can selectively label cathepsin B in eighteen cancer cell lines tested, making this ABP highly suitable for other biological setups. Moreover, using Cy5-labelled MP-CB-2 we were able to demonstrate by fluorescence microscopy that in cancer cells cathepsins B and L share overlapping, but not identical subcellular localization.

## Abstract 2

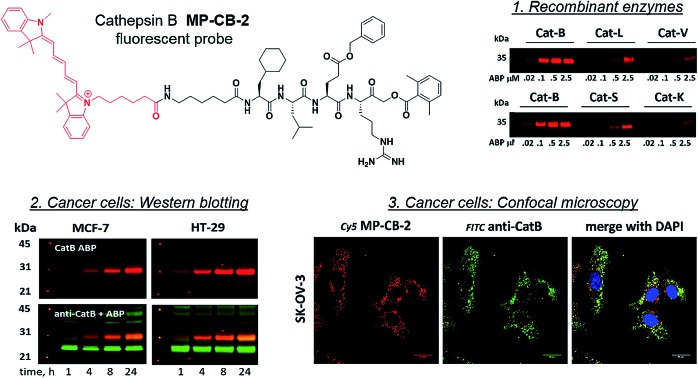
Highly selective fluorescent activity-based probe for the visualization of cathepsin B in cancer cells.

## 1. Introduction

Cysteine cathepsins are a structurally-related group of enzymes belonging to the papain superfamily of proteases (C1 family, CA clan in the Merops nomenclature).^1^,^2^ These enzymes tend to be optimally active in reducing, acidic conditions, typified by those found in the endolysosomal compartment, where they degrade proteins, thereby maintaining cellular homeostasis,^3^ however, over the past few decades the roles of cathepsins expanded and now it is evident that these enzymes are assigned to more specific functions including lipid metabolism, antigen presentation, autophagy, senescence/ageing and cellular stress signaling.^4^–^7^ Their localization is not only limited to acidic vacuoles, as active forms of these enzymes are also found in the cytosol, cell surface and in the extracellular space.^2^ Because of their essential role in maintaining cellular homeostasis, cathepsins are important players in systemic human diseases such as cancer, cardiovascular disorders, autoimmune diseases and osteoporosis.^8^–^12^

In cancer, elevated activities of cathepsins are associated with tumor development and progression, and poor patient prognosis in response to chemotherapy.^13^,^14^ As cathepsins are widely expressed and secreted into the extracellular milieu in a number of cancers by various tumor and tumor-associated immune cells, they are very attractive molecular targets for theranostic applications.^15^–^17^ The contribution of cathepsins to cancer progression is mainly due to the processing of extracellular matrix proteins, chemokine processing and shedding of cell surface molecules thereby enabling the cancer cells to migrate and invade other organs.^18^–^20^ However, it has been demonstrated that the activity of intracellular cathepsins may contribute to tumorigenesis, as inhibition of intracellular cathepsins resulted in increased tumor cell death and a reduced tumors size in several mice models.^21^

Among the cathepsins, cathepsin B, has gained much attention due to its attributed role in cancer progression.^22^ Its elevated activity has been detected in multiple human cancer cells lines as well as in all stages of tumorigenesis, from initiation to angiogenesis, invasion and metastasis.^12^,^15^,^22^–^24^ Cathepsin B is synthesized as a preproenzyme that is processed to its active form by the removal of the propeptide *via* acid-mediated auto-processing or by other lysosomal proteases, leading to mature cathepsin B.^25^ Despite major progress in the understanding of cathepsin B biology, its exact role in the tumor microenvironment has remained unclear. This is largely a consequence of the multitude of proteases that interact with each other or with other protein molecules including other enzymes, in a very complex fashion, thereby affecting numerous signaling pathways.^26^ Therefore, selective labeling of cathepsin B as well as other protease activities has the potential to unveil their functions in this complex environment.

The most convenient approach to detect or inhibit active cathepsins and other proteases, is to use fluorescent peptide substrates, inhibitors or activity-based probes tagged with a detectable moiety.^27^–^31^ However, since cathepsins display highly similar substrate preferences, it is very challenging to discriminate between them using peptide based reagents containing only natural amino acids.^32^,^33^ The specificity of cathepsins towards short substrates is mainly governed by P2–S2 interactions, these interactions are key determinants in achieving selectivity for individual cathepsins. So far, the most useful substrate for cathepsin B detection contains an Arg–Arg dipeptide at the P2 and P1 positions, since cathepsin B is the only cathepsin that tolerates Arg at the P2 position.^32^,^34^ Nevertheless, this substrate is hydrolyzed substantially slower in comparison with the general cathepsin substrate containing Phe–Arg residues at the same positions. However, this Phe–Arg sequence is also recognized by cathepsin L.^35^

The rapid progress in profiling protease specificity using various substrates and inhibitors has demonstrated that the protease active site can be successfully explored with a broad range of non-canonical (unnatural) amino acids, thereby largely expanding the available chemical combinatorial space used in their design. Using this concept, closely related enzymes can be discriminated by either^1^ dissecting small differences in specificity by structurally similar amino acids or^2^ by applying very large amino acids that can induce new interactions inaccessible for natural amino acids. Currently the most successful method, in terms of broad application and convenient chemical synthesis, for screening proteases with unnatural amino acids is HyCoSuL (Hybrid Combinatorial Substrate Library).^36^

In this work, we used two tailored chemical libraries to dissect the cathepsin B preferences at the P4–P1 positions. First, we profiled the enzyme with Ac-Ala-Arg-Leu-X-ACC library containing over 100 unnatural amino acids to determine the P1 specificity.^37^ This library was designed on specificity profiles of all cathepsins described by Choe *et al.*^32^ Next, we utilized the P1-Arg HyCoSuL library to determine cathepsin B preferences at the P4 to P2 region, which served as a basis for design of cathepsin B-selective substrates. The most selective substrates were then converted into AOMK-based inhibitors and decorated with Cy5/Cy7 fluorescent dyes to obtain highly-selective cathepsin B ABPs. Finally, we demonstrated that MP-CB-2 probe selectively labeled cathepsin B in a panel of eighteen human cancer cell lines, making this tool highly suitable for further biological studies.

## 2. Experimental section

### 2.1. Chemical reagents

All chemicals used for the synthesis of substrates and ABP were purchased from commercial suppliers and used without purification unless otherwise noted. The Rink amide RA resin (loading 0.48 mmol g^–1^) was used for the synthesis of ACC-labeled substrates, and the 2-chlorotrityl chloride resin (1.59 mmol g^–1^, 100–200 mesh) was used for the synthesis of peptides that were further converted into ABPs (both resins were from Iris Biotech GmbH, Germany). Fmoc-protected amino acids (all >98% pure) were purchased from various suppliers: Iris Biotech GmbH, Creosalus, P3 BioSystems, QM Bio, Bachem. *N*,*N*-Diisopropylethylamine (DIPEA, peptide grade), diisopropylcarbodiimide (DICI, peptide grade), piperidine (PIP, peptide grade), and trifluoroacetic acid (TFA, purity 99%) were all from Iris Biotech, GmbH. 2,4,6-Trimethylpyridine (2,4,6-collidine, peptide grade), triisopropylsilane (TIPS, purity 99%), 2,2,2-trifluoroethanol (TFE), anhydrous tetrahydrofuran (THF), hydrogen bromide (30% wt in AcOH), 4-methylmorpholine (NMM), isobutylchloroformate (IBCF), and 2,6-dimethylbenzoic acid (2,6-DMBA) were all purchased from Sigma Aldrich. *N*-Hydroxybenzotriazole (HOBt, monohydrate) was from Creosalus. HATU and HBTU (both peptide grade) were from ChemPep Inc. *N*,*N*′-Dimethylformamide (DMF, peptide grade) and acetonitrile (ACN, HPLC pure) were from WITKO Sp. z o.o., Poland. Methanol (MeOH, pure for analysis), dichloromethane (DCM, pure for analysis), diethyl ether (Et_2_O, pure for analysis), acetic acid (AcOH, 98% pure) and phosphorus pentoxide (P_2_O_5_, 98% pure) were from POCh, Poland. Fluorescent tags (cyanine-5 NHS and cyanine-7 NHS) were purchased from Lumiprobe. Diazomethane was generated according to the Aldrich Technical Bulletin (AL-180) protocol. Protease inhibitors E-64, E-64d, 3,4-DCI (3,4-dichloroisocoumarin), Bortezomib®, CA074, and CA-074Me were purchased from Sigma-Aldrich.

### 2.2. Preparation of recombinant human cathepsins B, L, V, S and K

The description for expression and purification of these proteases was published previously.^38^,^39^ All cathepsins were active-site titrated using E-64, a broad spectrum inhibitor.

### 2.3. Enzymatic kinetic studies

All kinetic experiments were performed using a CLARIOStar (BMG LABTECH) plate reader operating in fluorescence kinetic mode using 96-well plates (Corning®, Costar®). ACC-labeled fluorescent substrates were screened at 355 nm (excitation) and 460 nm (emission) wavelengths (gain 650). The cathepsin assay buffer was composed of 100 mM sodium acetate, 100 mM sodium chloride, 10 mM DTT, 1 mM EDTA, pH 5.5. All kinetic studies were performed at 37 °C.

### 2.4. Characterization of cathepsin B P4–P2 substrate specificity using HyCoSuL

To determine the cathepsin B specificity in the P4–P2 positions, we used the P1-Arg HyCoSuL library.^36^,^40^ P4, P3, and P2 sub-libraries were each screened at 100 μM final concentration in 100 μL final volume with human recombinant cathepsin B. The active cathepsin B concentration was in the 0.5–2 nM range, depending on the sub-library used. Total screening time was 30 min, but to avoid substrate depletion, only the linear portion of the progression curve (10–15 min) was used for analysis (RFU/s calculations). Screening of each library was performed a minimum of three times, and the average value was used to create the cathepsin B specificity matrix (S.D. for each substrate was below 15%). The hydrolysis rate for the best recognized amino acid at each position was set to 100%, and other amino acids were adjusted accordingly.

### 2.5. Characterization of cathepsin B specificity in the P1 position

To determine the cathepsin B specificity in the P1 position, we used the Ac-Ala-Arg-Leu-P1-ACC fluorogenic substrate library containing 19 natural and over 100 unnatural amino acids.^37^ The library was screened against cathepsin B at 4 μM final concentration in triplicate. Assay time was 30 min, but only the linear portion of substrate hydrolysis (5–15 min) was used for the analysis. The average value for each substrate was then calculated (S.D. for each substrate was below 10%). The Ac-Ala-Arg-Leu-Arg-ACC substrate served as a control, and its hydrolysis rate (RFU/s) was set as 100%, and other values were normalized accordingly.

### 2.6. Synthesis and screening of individual optimized substrates

ACC-labeled tetrapeptide substrates were synthesized and purified as described elsewhere.^41^ All potentially selective cathepsin B substrates were screened for their selectivity against five human cathepsins B, L, V, S and K (substrate concentration 100 μM, cathepsin concentration 5 nM). Substrate hydrolysis was carried out for 5 min for cathepsin B and up to 60 min for other cathepsins. Each experiment was performed in triplicate and the rate of substrate hydrolysis (RFU/s) is presented as an average value (S.D. for each substrate hydrolysis was below 15%).

### 2.7. Determination of kinetic parameters (*k*_cat_, *K*_M_, *k*_cat_/*K*_M_) of substrate hydrolysis

The kinetic parameters for all ACC-labeled substrates were determined using Michaelis–Menten kinetic according to the protocol described by Poreba *et al.*^41^ Because all tested substrates displayed a high degree of selectivity towards cathepsin B, the other four cathepsins were used at higher concentrations: cathepsin B: 0.3–0.5 nM, cathepsin L: 250 nM, cathepsin V: 200 nM, cathepsin S: 150 nM, and cathepsin K: 320 nM. All experiments were performed at least in triplicate and the results are presented as average values (S.D. for all tested substrates was below 15%).

### 2.8. Synthesis of fluorescent ABPs

Cy7- and Cy5-labeled irreversible ABPs probes for cathepsin B were synthesized and purified as described previously for cathepsin L by Poreba *et al.*^42^ In brief, Boc-(linker)-peptide-COOH was synthesized on solid support using 2-chlorotrityl chloride resin and used without further purification. In parallel, the Boc-Arg(Boc)_2_-AOMK warhead was synthesized through generation of diazomethane and reaction with mixed anhydrides (Boc-Arg(Boc)_2_-OH → Boc-Arg(Boc)_2_-CH_2_N_2_), following by the conversion of the crude product into bromomethyl ketone (Boc-Arg(Boc)_2_-BMK) and, finally, into acyloxymethyl ketone (Boc-Arg(Boc)_2_-AOMK). Next, the crude product was de-protected in TFA/DCM mixture, and the NH_2_-Arg-AOMK was coupled with Boc-(linker)-peptide-COOH to yield Boc-(linker)-peptide-Arg-AOMK. The product was purified on HPLC, and after Boc de-protection, it was labeled with Cy5 or Cy7 to obtain Cy5(Cy7)-(linker)-peptide-Arg-AOMK. The final product(s) were purified on HPLC, analyzed *via* HR-MS and HPLC and dissolved in DMSO (10 mM).

### 2.9. Determination of kinetic parameters for inhibition (*k*_obs_/*I*) of cathepsins

The kinetic parameters of inhibition of three irreversible Cy5/Cy7 labeled ABPs were determined for cathepsins B, L, V, S and K, and the inhibition parameters of CA-074 and CA-074Me were determined for cathepsins B and L. The *k*_obs_/*I* were measured under pseudo-first order reaction conditions. Individual cathepsins were mixed with various concentrations of ABP (at least 5-fold excess over cathepsins) and 100 μM ACC fluorescent substrate. Experiments were repeated at least in triplicate, and the results are presented as an average (S.D. were below 20%).

### 2.10. Antibodies

Primary anti-hCathepsin antibodies were purchased from commercial suppliers and used as indicated below. Goat anti-hCathepsin L (R&D, AF952, Western Blot (WB): 0.1 μg mL^–1^, ImmunoFluorescence (IF): 5 μg mL^–1^); goat anti-hCathepsin B (R&D, AF953, WB: 0.2 μg mL^–1^, IF: 5 μg mL^–1^), mouse anti-hCathepsin V (R&D, MAB1080, WB: 1 μg mL^–1^), goat anti-hCathepsin S (R&D, AF1183, WB: 0.2 μg mL^–1^), mouse anti-hCathepsin K (Santa Cruz Biotechnology, E-7, sc-48353, 2 μg mL^–1^). Primary anti-hCathepsins were detected with fluorescent labeled secondary antibodies, Western Blot: IRDye® 800CW, donkey anti-Mouse (1 : 10 000, LI-COR) or IRDye® 800CW, donkey anti-Goat (1 : 10 000, LI-COR), ImmunoFluorescence: Alexa Fluor™ 488 donkey anti-Goat IgG (H + L) (A11055, 1 : 300, Life Technologies).

### 2.11. Cell culture

In this study we used eighteen human cancer cell lines (all purchased from the American Type Culture Collection, ATCC). Jurkat T cells (*peripheral blood; acute T cell leukemia*), THP-1 cells (*peripheral blood; acute monocytic leukemia*), U-266 (*peripheral blood; myeloma*) K-562 (*bone marrow, chronic myelogenous leukemia – CML*), NCI-H226 cells (*lung; squamous cell carcinoma*), U-937 (*lymphocyte; histiocytic lymphoma*) were cultured in RPMI-1640 medium supplemented with 10% Fetal Bovine Serum (FBS), 2 mM of l-glutamine, 100 units per mL penicillin and 100 μg per streptomycin. MDA-MB-231 (*mammary gland/breast; adenocarcinoma*), MCF-7 (*mammary gland/breast; adenocarcinoma*), OVCAR-5 (*ovary, adenocarcinoma*), A-431 (*skin/epidermis; epidermoid carcinoma*), PC-3 (*prostate, adenocarcinoma*), HeLa (*cervix, adenocarcinoma*) and SH-SY5Y (*bone marrow, neuroblastoma*) were cultured in Dulbecco's Modification of Eagle's Medium (DMEM) supplemented with 10% FBS, 2 mL of l-glutamine and penicillin/streptomycin (as described above). HT-29 (*colon; colorectal adenocarcinoma*), HCT-116 (*colon; colorectal carcinoma*), U-2 OS (*bone, osteosarcoma*), Saos-2 (*bone, osteosarcoma*) and SK-OV-3 (*ovary, adenocarcinoma*) were cultured in McCoy's 5A medium supplemented with 10% FBS, 2 mL of l-glutamine and penicillin/streptomycin (as described above) (Saos-2 cells were cultured in 15% FBS/McCoy's 5A medium). Adherent cells were detached using trypsin-EDTA (0.25%), phenol red solution.

### 2.12. Detection of cathepsins in cancer cells using specific antibodies

All cancer cells were cultured on 150 mm cell culture Petri dishes (30 mL) to 90% confluence (adherent cells) or to a density above 2 × 10^6^ cells per mL (non-adherent cells). Next, the cells were harvested, centrifuged at 500 × *g* for 5 min, washed with 1× PBS (phosphate-buffered saline), counted, and centrifuged again at 500 × *g* for 5 min. The cell pellet was then lysed in RIPA buffer (volume was adjusted to obtain 5 × 10^6^ cells per mL) for 45 min on ice and vortexed occasionally. Next, cell lysates were sonicated and centrifuged at 14 000 × *g* for 15 min at +4 °C. Each supernatant was collected and total protein concentration was calculated based on *A*_280_. All cell lysates were then adjusted (with RIPA buffer) to the final protein concentration of 1 mg mL^–1^. Next, 20 μL of each cell lysate was subjected to SDS-PAGE analysis (4–12% Bis–Tris gel, 200 V, 30 min) followed by membrane transfer (0.2 μm nitrocellulose membrane, 10 V, 60 min). Ponceau S staining verified equal loading and successful transfer of proteins. Next, each membrane was blocked in 2.5% BSA in TBS-T for 1 hour at room temperature. Primary anti-human cathepsin antibodies (in 1% BSA in TBS-T) were incubated separately with the membranes overnight at +4 °C at the concentrations described in the “antibodies” section. In the next step, secondary antibodies were incubated with the membranes for 30 min at room temperature as described in the “antibodies” section. Membranes were then scanned at 800 nm using the LI-COR system. Each cell lysate was used to detect five cathepsins (B, L, V, S, and K). In parallel to antibodies staining, we used cancer cell lysates to demonstrate equal protein loading. For this purpose, we performed SDS-PAGE analysis (as described above) and stained all the proteins on a gel using instant blue (4 hours incubation at room temperature).

### 2.13. Detection of active cathepsin B in cancer cell lysates using Ac-Cha-Leu-Glu(*O*-Bzl)-Arg-ACC substrate

Cell pellets from 50 000 cells were re-suspended in cathepsin assay buffer containing 0.5% Triton X-100 for cell lysis. Cell lysis was performed on ice for 30 min. After this time, cells were centrifuged at max speed for 5 min, and the supernatant (supplemented with 10 mM DTT) was used for the kinetic assay. In each assay 100 μM of cathepsin B selective substrate Ac-Cha-Leu-Glu(*O*-Bzl)-Arg-ACC was used, and the reaction progress was monitored for 30 min. In the experiments with inhibitors, cell lysate was pre-incubated with inhibitor for 15 min, followed by substrate addition and kinetic readout. Each measurement was performed three times and the substrate hydrolysis rate (RFU/s) in control (no inhibitor) was set to 100%, and other values were adjusted accordingly.

### 2.14. Detection of active cathepsin B in cancer cells using Cy5-labeled MP-CB-2 probe

50 000 cells from fourteen adherent cancer cell lines were seeded into 12-well plates and allowed to attach overnight. In parallel, 50 000 cells from four non-adherent cancer cell lines were placed into 12-well plates and cultured overnight. The next day, MP-CB-2 ABP was added to the cells (1 μM final concentration) and incubated for various times (1, 4, 8, 24 hours). Next, cells were harvested, centrifuged at 500 × *g* for 5 min, cell pellets were washed with 1× DPBS, cells were centrifuged again at 500 × *g* for 5 min, and the supernatant was discarded. Finally, cell pellets were solubilized in 100 μL of 1× SDS/DTT and boiled for 5 min, cooled to room temperature and sonicated. Each sample (30 μL) was then subjected to SDS-PAGE analysis (4–12% Bis–Tris Plus 10-well gels, 200 V, 30 min), followed by protein transfer onto the membrane (0.2 μm nitrocellulose membrane, 10 V, 60 min). Ponceau S staining verified equal loading and transfer of proteins. Membranes were blocked in 2.5% BSA in TBS-T for 1 hour at room temperature. Primary anti-human cathepsin B antibody (in 1% BSA in TBS-T) was incubated with the membranes overnight at +4 °C. Secondary antibody (IRDye® 800CW, donkey anti-Goat) was incubated with the membranes for 30 min at room temperature as described in the “antibodies” section. The membranes were then scanned at 700 nm (red channel for Cy5 detection) and 800 nm (green channel for 800CW antibody) using an Odyssey imaging system (LI-COR). The images were then analyzed with the Image Studio software.

### 2.15. Detection of active cathepsin B in selected cancer cells using MP-CB-2 probe

50 000 of selected cancer cells (A-431, HCT-116, OVCAR-5, SK-OV-3, MDA-MB-231, U2-OS, and PC-3) were seeded into 12-well plates and allowed to attach overnight. The next day, MP-CB-2 ABP (1 μM final concentration) was added to the cells and incubated for various times (none, 1, 2, 4, 8, 24 hours). Control cells were preincubated with E-64d (25 μM) for 2 hours, and then incubated with MP-CB-2 probe for 24 hours. Next, cells were harvested and prepared for SDS-PAGE analysis as described in the above section. The probes covalently label the catalytic Cys. Since this is located in the light chain of cathepsin B we would expect to label:^1^ light chain alone, and^2^ single chain (light chain + heavy chain). In order to detect the light chain of cathepsin B (6 kDa), electrophoresis was run for only 25 min (4–12% Bis–Tris Plus 10-well gels, 200 V) in the presence of 2 μL of PageRuler™ Prestained Protein Ladder in order to visualize low molecular weight protein migration (green band, 10 kDa). Next, proteins were transferred onto the membrane (0.2 μm nitrocellulose membrane, 10 V, 60 min) and Ponceau S was used to verify equal loading and transfer. Membranes were then blocked in 2.5% BSA in TBS-T for 1 hour at room temperature. All SDS-PAGE analyses and protein transfers were duplicated as one membrane was incubated with goat anti-human cathepsin B antibody and the second one was incubated with goat anti-human cathepsin L antibody (see “antibodies” section). Finally, the membranes were incubated with a secondary antibody (IRDye® 800CW, donkey anti-Goat) for 30 min at room temperature, and scanned at 700 nm and 800 nm to detect the Cy5 probe and cathepsins-B/-L antibodies. All images were analyzed with Image Studio software.

### 2.16. Detection of cathepsin B in cancer cells by immunofluorescence

15 mm glass coverslips were placed in 12-well plates and coated with poly-l-lysine (500 μL, 0.01% in PBS, R&D Systems) for 30 min at room temperature. Poly-l-lysine was aspirated and coverslips were washed with sterile water (twice; 1 mL each) and air-dried for 60 min. Next, low passage A-431 cells in DMEM (50 000 cells per mL) were added to the wells (1 mL per well) and allowed to attach overnight. Cells were then treated with Cy5-labeled MP-CB-2 probe (1 μM) for 24 hours. Control cells were preincubated with E-64d (25 μM) or with CA-074Me (25 μM) for 4 hours prior to the addition of the MP-CB-2 probe. Next day, the medium was removed and the cells were washed with 1× DPBS (twice; 1 mL each). Cells were then fixed with ice-cold methanol (1 mL per well) for 15 min at –20 °C. Methanol was gently aspirated, cells were washed with 1× PBS (twice, 1 mL each), blocked and permeabilized with 5% BSA in 1× DPBS (with 0.3% TritonX-100, v/v) for 1 hour at room temperature, and washed twice with 1% BSA in DPBS (with 0.3% TritonX-100, v/v). Cathepsins B and L were detected by primary goat anti-human antibodies (see the “antibodies” section) in 1% BSA in DPBS (with 0.3% TritonX-100, v/v) overnight at +4 °C. The next day, antibodies were aspirated, and cells were washed twice with 1% BSA in DPBS (with 0.3% TritonX-100, v/v) and labeled with secondary antibody (488 AlexaFluor™, see “antibodies” section) in 1% BSA in DPBS (with 0.3% TritonX-100, v/v) for 1 hour at room temperature. Next, the cells were washed twice with DPBS, coverslips were mounted with Vectashield fluorescence mounting medium containing DAPI (Vector Lab. H-1000) on Superfrost Plus microscope slides (Fisher Scientific, no. 12-550-15), and sealed with nail polish. Slides were stored at +4 °C until use. Cells were then subjected to confocal microscope analysis using a Zeiss microscope (LSM 710 NLO, objective 63×/1.4 DIC oil). DAPI was detected by the UV channel (Mai-Tai Laser HB 690–1020 nm), the Cy5 MP-CB-2 probe was detected with the Cy5 filter (single photon laser: 633 nm), and cathepsin/secondary antibodies were read using the FITC filter (single photon laser: 458 nm). All images were acquired in .lsm format using ZEN software and analyzed with ImageJ software. Images shown are representative views of cells from two coverslips. Detection of cathepsin B in other cancer cells (HCT-116, OVCAR-5, SK-OV-3, MDA-MB-231, U2-OS, and PC-3) was performed in the same way.

### 2.17. Weighted colocalization coefficients

To quantify ABP/cathepsin colocalization we calculated weighted colocalization coefficients between the MP-CB-2 ABP (red) and cathepsin B (green) and between the MP-CB-2 ABP (red) and cathepsin L (green) using ZEN 2011 software. These calculations were performed by summing the pixels (red and green) in the colocalized regions (whole images) and dividing by the sum of the pixels in the red channel (ABP). The value of each pixel was equal to its intensity value (from 0 to 1), thus a weighted colocalization coefficient is more accurate here than the standard colocalization coefficient where all the pixels, regardless of their intensity, have a value of 1. To eliminate the red and green staining background in these calculations, we set crosshairs according to single label controls as described elsewhere.^43^ To enhance accuracy, for each cell line investigated, we calculated the weighted colocalization coefficient based on the analysis of at least randomly selected seven fluorescent images.

### 2.18. Detection of cathepsin B in lung cancer patient's sample

Pleural effusion fluid from IV-stage non-small-cell lung cancer patient was obtained in clinical collection bag at Moores Cancer Center (University of California San Diego Health). The effusion was centrifuged at 300 × *g* for 25 minutes in 50 mL disposable conical tubes to separate tumor fragments from floating cells. The solid component was re-suspended in PBS and incubated with an enzymatic mixture consisting of 3 U collagenase and 24 U dispase, for 2–3 hours. Enzymes were refreshed every 60 minutes, and the liquid portion was decanted on ice following a one minute rest. The resultant cell mixture was strained through a 70 μm filter, and gradient separated using a Ficoll medium (Sigma-Aldrich), spun at 400 × *g* for 20 minutes. Cells were assessed for viability using Tryphan blue reagent and plated on 12-well plates. Plating medium consists of MCDB-1 (Thermo Fisher) with addition of the EGM-2 MV BulleKit (Lonza) and 10 mM l-glutamine (Corning). Cells were cultured for 3 weeks to reach 70% confluency. And the cathepsin B visualization *via* Western blotting and confocal fluorescence microscopy was performed as described above.

## 3. Results and discussion

### 3.1. Cathepsin B substrate specificity in the P4–P1 positions

**Fig. 1:**
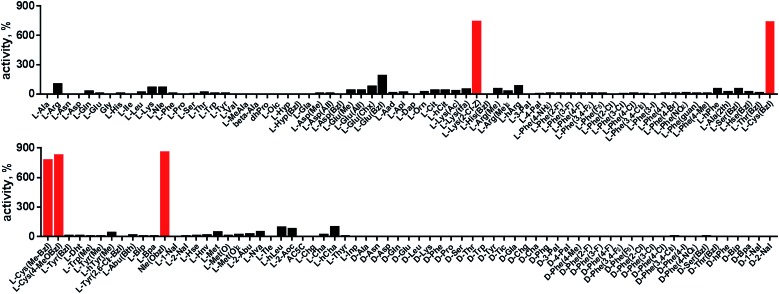
Human cathepsin B specificity in the P1 position. P1 preference of human cathepsin B was determined using Ac-Ala-Arg-Leu-P1-ACC fluorogenic substrate library containing 19 natural and over 100 unnatural amino acids. The *x* axis shows abbreviated amino acids, and the *y* axis displays relative activity of each substrate adjusted to Ac-Ala-Arg-Leu-Arg-ACC substrate (100%) which served as a control. The P1 specificity screening was performed in triplicate. The substrate hydrolysis (RFU/s) data are presented as an average (S.D. for each substrate was below 10%). The best recognized amino acids (Lys(2CL-Z), Cys(Bzl), Cys(MeBzl), Cys(Me)Bzl and Nle(OBzl)) are shown in red.

**Fig. 2:**
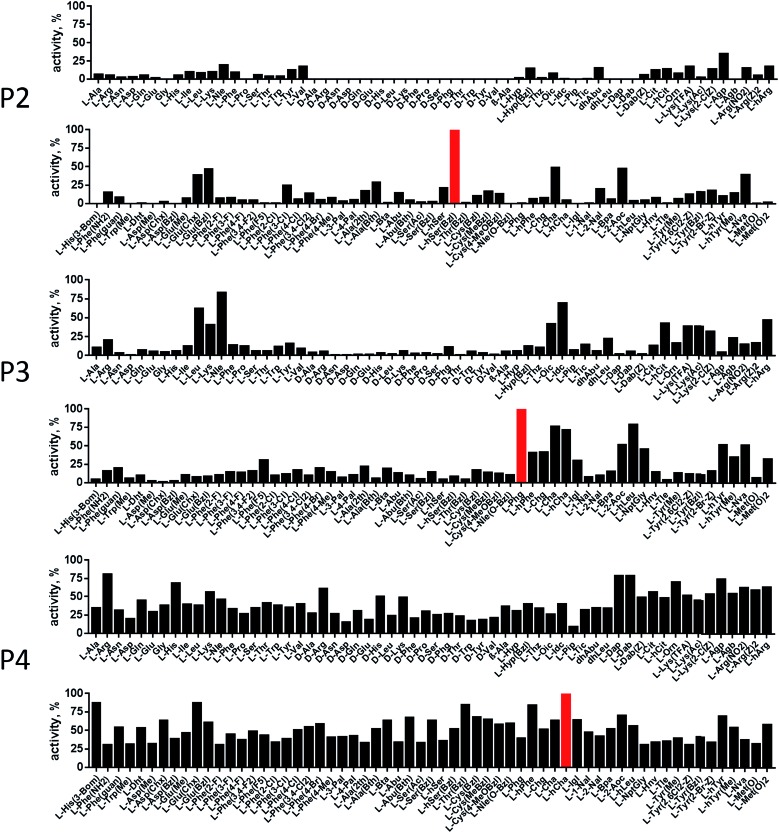
Human cathepsin B specificity in the P4–P2 positions. The P4–P2 preferences of human cathepsin B were determined using the P1-Arg HyCoSuL library. The *x* axis shows abbreviated amino acids, and the *y* axis displays relative activity of each substrate adjusted to the best recognized amino acid (marked in red; hSer(Bzl) in P2, Phg in P3, and hCha in P4). Each sub-library (P4, P3, P2) screening was performed in triplicate and the substrate hydrolysis data (RFU/s, %) are presented as average (S.D. for each substrate was below 10%).

In order to develop selective substrates and ABPs for cathepsin B, we profiled its specificity in the P4–P1 positions using a wide range of unnatural amino acids. To gain insight on cathepsin B P1 preferences, we used the previously published Ac-Ala-Arg-Leu-P1-ACC library, that was designed based on lysosomal cathepsin specificity profiles.^32^ Using this library, we demonstrated that Arg is the best recognized natural amino acid in the P1 position. However, some unnatural derivatives were hydrolyzed 7–9 fold faster than Arg (Fig. 1). These were bulky and hydrophobic derivatives of Lys and Cys, namely Lys(2-Cl-Z), Cys(Bzl), Cys(Me-Bzl), Cys(MeOBzl) and Nle(OBzl). A comparison of cathepsin B and cathepsin L substrate specificity in the P1 position revealed that both proteases have a highly conserved S1 pocket, displaying similar substrate preferences (Fig. S1†). These results clearly demonstrate that cathepsin B S1 pocket can accommodate positively charged Arg and Lys, but also large and hydrophobic unnatural derivatives. To get a further insight into cathepsin B substrate preferences, we screened the enzyme preferences in the P4–P2 positions using the HyCoSuL library with fixed Arg in the P1 position (Fig. 2).^40^,^42^ This library is composed of three sublibraries (P4: Ac-X-mix-mix-Arg-ACC; P3: Ac-mix-X-mix-Arg-ACC; and P2: Ac-mix-mix-X-Arg-ACC; where mix is an equimolar mixture of 19 natural amino acids, omitting cysteine and substituting norleucine for methionine, and ACC is a fluorescent tag) and contains over 100 unnatural amino acids in each position. The screening data allowed us to make a cathepsin B broad specificity profile around S4–S2 active site pockets (Fig. 2). Cathepsin B specificity in the P4–P2 positions for natural amino acids was in line with previously published data,^32^ however, the use of unnatural amino acids enabled us to explore this chemical space more precisely. We found that cathepsin B has a preference for large, hydrophobic amino acids (hSer(Bzl), Glu(Bzl), hCha) in the P2 position, which were significantly better recognized (>5-fold) than the best natural amino acid, valine. This observation is in line with the current understanding of cathepsin specificity that the P2–S2 interactions are crucial for substrate recognition. More importantly, our data demonstrate that large unnatural amino acids, in contrast to natural analogues, can induce new interactions around the protease active site that enhance substrate processing. The analysis of the P3 position revealed that the best accepted amino acids were those with aliphatic side chains (Leu, Nle, hCha, 2Aoc, hLeu, Oic). However, amino acids from other groups (basic and Phe-derivatives) were also well accepted. The analysis of the P4 position demonstrated that cathepsin B has almost no specificity in the S4 pocket, as even amino acids with d-stereochemistry were tolerated.

### 3.2. Cathepsin B selective substrates

**Fig. 3:**
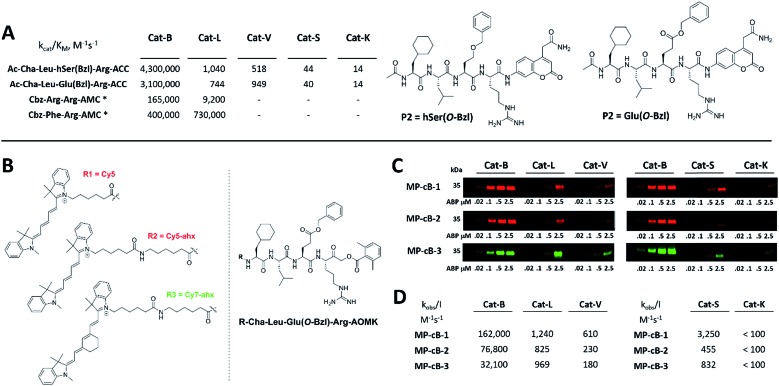
Selective substrates and ABPs for cathepsin B. (A) The structures and kinetic parameters of hydrolysis (*k*_cat_/*K*_M_)^1^ for the most selective cathepsin B ACC-labeled substrates developed through HyCoSuL profiling and^2^ for two reference substrates (*): cathepsin B selective (Cbz-Arg-Arg-AMC) and broad spectrum (Cbz-Phe-Arg-AMC).^35^ (B) Structures of three cathepsin B potent and selective ABPs containing the Cha-Leu-Glu(Bzl)-Arg sequence. Each ABP is labeled with either Cy5 or Cy7 fluorescence tag. (C) Labeling of recombinant cathepsins using three cathepsin B selective ABPs. Enzymes (100 nM final concentration) were incubated separately with various probe concentrations for 30 min and then subjected to SDS-PAGE analysis. Fluorescence was scanned using the 700 nm channel (Cy5) or 800 nm channel (Cy7) (LI-COR instrument). (D) The inhibition constant (*k*_obs_/*I*) of three cathepsin B ABPs determined for the five recombinant cathepsins.

HyCoSuL screening provided information about cathepsin B specificity, however, the presence of unnatural amino acids in the peptide library allows for the opportunity to design substrates that can distinguish between closely related proteases. Since cathepsins B and L are the most abundant lysosomal cathepsins, we attempted to individualize each enzyme with tetrapeptidic substrates containing unnatural amino acids. To do this, we compared our previously published cathepsin L profile with the cathepsin B profile (Fig. S2†).^42^ This comparison uncovered the main differences between these enzymes and guided us towards the 1^st^ generation of cathepsin B-selective substrates (Table S1 and Fig. S3†). After kinetic screening, these peptides were optimized to obtain the 2^nd^ generation of substrates that were extremely selective towards cathepsin B over cathepsins L, V, S, and K (Fig. S3 and Table S2†). Generally, these substrates contained aliphatic amino acids in the P4/P3 positions; bulky hydrophobic amino acids (hSer(Bzl), Glu(Bzl) or Glu(Chx)) in the P2 position, and Arg in the P1 position. The most selective cathepsin B substrate (Ac-Cha-Leu-Glu(Bzl)-Arg-ACC) displayed over 3500-fold selectivity over cathepsin L, whereas the most commonly used commercial cathepsin B selective substrate (Cbz-Arg-Arg-AMC) is only 17-fold more selective for cathepsin B over cathepsin L (Fig. 3A and Table S3†).

### 3.3. Design and analysis of cathepsin B selective ABPs

The cathepsin B selective peptides were further used to design fluorescently labeled ABPs. To do this, we utilized the Cha-Leu-Glu(Bzl)-Arg sequence and added either a Cy5 or Cy7 fluorescent tag. We also added a ahx linker to both probes to separate the fluorophore from the peptide. We selected the acyloxymethyl ketone group (AOMK) to use as the warhead, as this electrophile is known to covalently react with the catalytic cysteine residue of the cathepsins (Fig. S4†).^32^ The structures of all three cathepsin B probes are shown in Fig. 3B. Next, to determine the selectivity of the probes, we performed *in vitro* labeling using five human recombinant cathepsins (B, L, V, S and K). ABPs were incubated at various concentrations (20 nM, 100 nM, 500 nM, and 2.5 μM) with a 100 nM final concentration of each active cathepsin for 30 min, followed by SDS-PAGE. The gels were then scanned for the fluorescent signal generated by labeling of the enzyme with the appropriate ABP (Fig. 3C). MP-CB-1 was the most potent cathepsin B probe, as the labeling signal was almost saturated at the probe/enzyme ratio 1 : 1. However, this probe displayed cross-reactivity with cathepsins L and S when used at high concentrations (2.5 μM, probe/enzyme 25 : 1 ratio). Modification of the MP-CB-1 probe by adding an ahx linker (MP-CB-2) retained potency and reduced the off-target labeling of cathepsins L and S. However, when the Cy5 tag was exchanged for Cy7 (MP-CB-3), the probe lost both potency and selectivity (Fig. 3C). To get a better insight into the probe kinetics we measured their *k*_obs_/*I* parameters towards five cathepsins (Fig. 3D). This kinetic data reflects the *in vitro* labeling results, as the most potent cathepsin B probe was MP-CB-1 (*k*_obs_/*I* = 162 000 M^–1^ s^–1^) and the most selective was MP-CB-2. As shown by the labeling experiment the Cy7-labeled MP-CB-3 probe was less potent than the Cy5-derivatives. As the MP-CB-2 displayed both high cathepsin B potency and selectivity in comparison to other cathepsins, we selected this probe for the detection of cathepsin B in living cells. However, it must be stressed here, that although the optimal cathepsin B ACC-substrate and AOMK activity-based probe share the same peptide sequence, the ABP is less selective (*k*_obs_/*I* parameter), than the fluorescence substrate (*k*_cat_/*K*_M_ parameter). The main reason is, that in contrast to ACC substrates, the reactive AOMK electrophile group reacts with proteases in a covalent and irreversible fashion. Therefore after prolonged incubation more protease-ABP complexes are formed, which results in off-target labeling.^43^–^45^

### 3.4. Cathepsin detection in cancer cell lysates

**Fig. 4:**
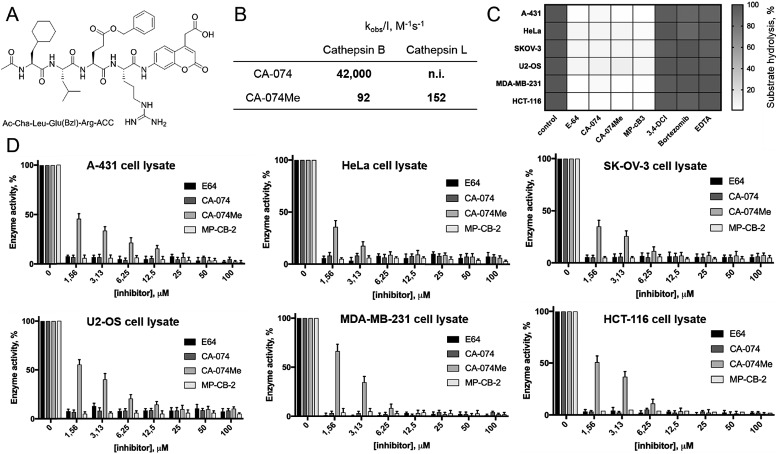
Detection of cathepsin B activity is cell lysates using fluorescent substrate. (Panel A) The structure of cathepsin B selective fluorogenic substrate used in this study. (Panel B) The potency (*k*_obs_/*I*) of two widely used cathepsin B inhibitors (CA-074 and CA-074Me) towards human recombinant cathepsins B and L. (Panel C) The inhibition profile of cathepsin B activity in selected cell lysates at 100 μM of protease inhibitors. (Panel D) The inhibition profile of cathepsin B activity in selected cell lysates as a function of various inhibitors concentrations.

Since cathepsin activity levels increase in tumor cells, they have long been recognized as good biochemical markers for cancer detection, in both early stage of cancer development and during advanced stages including metastasis.^12^ One method for cathepsin detection is the use of selective ABPs that bind only to the active enzyme.^46^–^48^ Having optimized the structure of cathepsin B selective ABP we aimed to test its performance in multiple cancer cells lines with different levels of cathepsins. To investigate the abundances of individual cathepsins across cancer cells we first analyzed eighteen cells lines covering different types of cancer: leukemia (Jurkat T, THP-1), myeloma (U-266), lymphoma (U-937), neuroblastoma (SH-SY5Y), bone marrow cancer (K-562), bone cancer (U-2 OS, Saos-2), ovarian cancer (SK-OV-3, OVCAR-5), lung cancer (NCI-H226), breast cancer (MDA-MB-231, MCF-7), skin cancer (A-431), prostate cancer (PC-3), cervix cancer (HeLa) and colon cancer (HT-29, HCT-116). From each cell line, we prepared lysates and subjected them to SDS-PAGE, followed by Western blotting. Detection of cathepsins with antibodies uncovered an uneven distribution across different cancer cell lines (Fig. S5 and S6†). What is also important for detection of cathepsin activities is the observation that different cell lines display different extent of cathepsin processing (Fig. S5†). Interestingly, cathepsin B is the only one that contains the catalytic cysteine in the light chain (∼6 kDa, not detected with antibodies). Therefore we expected that both light chain and single chain (light chain + heavy chain) will be detected with our ABP. Cathepsin L exists in two catalytically active variants (single chain and heavy chain), and cathepsins V, S, and K have only one, the main chain (Fig. S5†). In order to validate our chemical approach for development of cathepsin B selective reagents, we tested our champion substrate (Ac-Cha-Leu-Glu(*O*-Bzl)-Arg-ACC) in selected cell lysates containing various amounts of cathepsin B and cathepsin L (Fig. 4). We selected cell lines with high cathepsin L and low cathepsin B expression (HeLa, A431, U2-OS), and with either low (HCT-116) or high (SKOV-3, MDA-MB-231) expression of both enzymes. We found that regardless of cell line used, we were able to selectively detect intracellular cathepsin B activity with our substrate. This selectivity was further confirmed by the use of cathepsin B selective inhibitors (CA074, and CB-MP-2), which completely depleted its catalytic activity. Moreover, we showed that CA-074Me is not selective towards cathepsin B, which is in line with the previous reports.^49^ Although CA-074Me appears as a very weak inhibitor towards recombinant cathepsin B in a test tube (Fig. 4B), its high inhibitory potency and selectivity in cell lysates might be explained by the fact that the methyl group is rapidly removed by esterases, leading to the formation of highly active CA-074 analogue (Fig. 4C and D).

### 3.5. Detection and selective labeling of cathepsin B in cancer cells

**Fig. 5:**
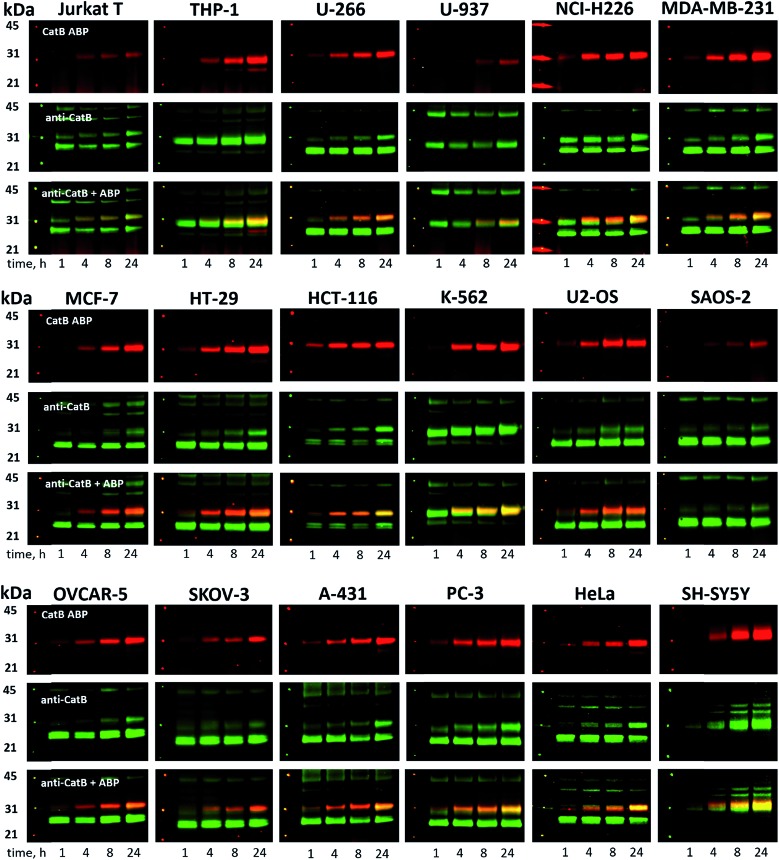
Cathepsin B labeling in human cancer cells using MP-CB-2 ABP. Eighteen human cancer cell lines were incubated with 1 μM Cy5-labeled MP-CB-2 ABP for various time (1, 4, 8, 24 hours), followed by SDS-PAGE and Western blot analysis. The red band indicates cathepsin B (29 kDa single chain form – active enzyme) labeling with probe, and the green band is the anti-cathepsin B antibody staining (∼43 kDa proenzyme, ∼29 kDa single chain and ∼26 kDa heavy chain). The MP-CB-2 probe is highly-selective towards cathepsin B and in most cell lines it can detect cathepsin B after 1 hour after incubation. After protein transfer to the membrane, molecular weight marker was marked with fluorescent ink to visualize it on both 700 nm (red) and 800 nm (green) channels.

Since cysteine cathepsins are involved in the invasion, metastasis, and proliferation of cancer cells they are attractive targets for theranostics, which integrates therapeutic interventions and diagnostic tools.^15^ Thus, our ultimate goal was to design a cathepsin B probe capable of selective inhibition and labeling of this enzyme in cells. As indicated in Fig. S5,† cathepsin B and other cathepsins are expressed in multiple types of cells making their individual detection a challenge that requires tools with high selectivity. Our initial *in vitro* labeling and kinetics indicate that cathepsin B can be distinguished from other lysosomal cathepsins by using MP-CB-2 ABP. To validate this, we incubated 1 μM probe for various time (1, 4, 8, 24 hours) separately with a panel of eighteen different cancer cell lines. Next, cells were harvested, subjected to SDS-PAGE and Western blot analysis and scanned at 700 nm channel (ABP) and 800 nm channel (cathepsin B antibody). After samples denaturation active cathepsin B can be detected in one of the two forms:^1^ ∼6 kDa light chain and^2^ ∼29 kDa single chain (light chain + heavy chain). The results demonstrated that in all tested cell lines the MP-CB-2 probe was taken up into cellular endolysosomal compartments and labeled cathepsin B very selectively even after prolonged (24 hours) incubations, as confirmed by co-labeling with anti-cathepsin B antibody (Fig. 5). The data also demonstrated that the probe uptake and the efficiency of cathepsin B labeling was strongly cell-dependent. In some cells MP-CB-2 probe was taken up rapidly (1 hour) and the saturation of labeling was seen after 4–8 hours (HCT-116, NCI-H226, U2-OS, HeLa), whereas in other cells the labeling was visible after 8 hours (U-937). Moreover, the cathepsin B labeling remained very selective even in cells with low cathepsin B expression and significant expression of cathepsins L and S (*i.e.* Jurkat T, THP-1, Saos-2, A-431) (Fig. 5, and S5†). This experiment indicates that regardless of the activity levels of particular cathepsins across different cell lines, the Cy5 labeled MP-CB-2 probe displayed high selectivity towards cathepsin B. However, in some cell lines MP-CB-2 ABP displayed some cross-reactivity with an unknown non-cathepsin protein target (Fig. S7†).

### 3.6. Detection of active cathepsin B by fluorescence microscopy

**Fig. 6:**
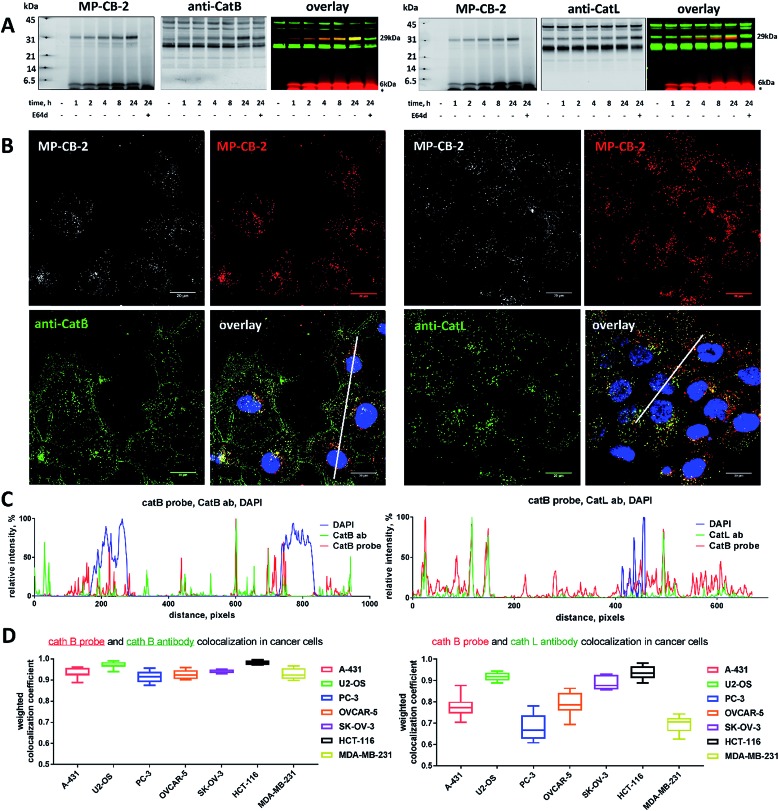
Cathepsin B localization in A-431 cell line. (A) Cathepsin B was selectively labeled in A-431 cell line using 1 μM MP-CB-2 ABP (left panel). This probe labeled single chain cathepsin B form (∼29 kDa) and also the light chain (∼6 kDa), which dissociated from the heavy chain after sample boiling. The cathepsin B labeling could be prevented using E-64d inhibitor. MP-CB-2 probe did not label cathepsin L even after prolonged incubation (24 hours). Color code for the overlay Western blot: MP-CB-2 probe is red, and cathepsin antibody is green. After protein transfer to the membrane, molecular weight marker was marked with fluorescent ink to visualize it on both 700 nm (red) and 800 nm (green) channels. (B) Localization of cathepsin B in A-431 cells using 1 μM of ABP (overnight incubation) and anti-cathepsin B antibody. ABP and catB antibody greatly overlapped, which demonstrates the ABP selectivity. However, the signal from the probe also overlapped with anti-cathepsin L antibody, demonstrating that both enzymes share the same subcellular localization. MP-CB-2 probe could also label nuclear cathepsin B. (C) The colocalization plot of cathepsin, Cy5 probe and nucleus from randomly selected area (line). Data were captured from a line drawn through the cell (white line on Panel B, overlay). The *y* axis shows the relative intensity of each signal, and the *x* axis displays the distance (in pixels). These plots demonstrate the high MP-CB-2 probe selectivity (left panel) and nuclear localization of cathepsin B (left panel). Scale bar is 20 μm. (D) Colocalization of cathepsin B MP-CB-2 ABP with cathepsin B (left) and cathepsin L (right) in seven cancer cell lines (A-431, U2-OS, PC-3, OVCAR-5, SK-OV-3, HCT-116, and MDA-MB-231) presented on box plots. The weighted colocalization coefficients were calculated for ABP/cat B and ABP/cat L based on the analysis of at least seven randomly selected images taken for each cell line.

Our data indicated that CB-MP-2 can selectively label cathepsin B in various cell lines even after prolonged (24 hours) incubation. Next, we used this probe to detect cathepsin B activity in cancer cell lines by florescence microscopy. We selected seven cell lines that display various levels of cathepsin expression (according to labeling in Fig. S5†): A-431 (low cathepsin B, and high cathepsins L and S), HCT-116 (low expression of all cathepsins), PC-3 (moderate expression of all cathepsins), OVCAR-5 (moderate expression of cathepsin B, and high expression of cathepsins L and V), U2-OS (high expression of cathepsins B and L), and MDA-MB-231 and SK-OV-3 (high expression of all cathepsins). Each of the selected cell lines were first incubated with the MP-CB-2 probe (1 μM) for various times (1, 2, 4, 8, 24 hours), followed by SDS-PAGE and Western blot analysis (Fig. 6A). We performed parallel experiments, where one membrane was stained with the anti-cathepsin B antibody, and the second one with the anti-cathepsin L antibody. We chose cathepsin L, since this enzyme is highly expressed in multiple cell lines, similar to cathepsin B, and shares some substrate preferences with cathepsin B. We demonstrated that cathepsin B labeling in A-431 (low cathepsin B level, high cathepsin L level) was very selective even after 24 hours, as confirmed by immunologic detection (Fig. 6A). As expected, we were able to label both cathepsin B active forms (single chain 29 kDa, and light chain 6 kDa). Moreover, the cathepsin B labeling could be completely prevented by pre-incubation with the cell-permeable broad spectrum cathepsin inhibitor (E-64d, 25 μM) or with cathepsin B cell-permeable inhibitor, CA-074Me (Fig. S8†). The selective detection of cathepsin B single chain and light chain were also demonstrated in other cell lines (Fig. S9A–S14A†). The cathepsin B labeling profiles provided us with the information on how long the CB-MP-2 probe can be incubated with cells in order to provide the desired selectivity. Next, we utilized this information to visualize cathepsin B in selected cell lines using confocal fluorescence microscopy. We incubated 1 μM MP-CB-2 probe with cells for 8 or 24 hours (depending on the cell type), and then methanol-fixed cells were labeled with either cathepsin B or cathepsin L antibodies for imaging. We found that in the A-431 cell line probe signal (red) overlapped with the cathepsin B antibody staining (green) (Fig. 6B). However, as indicated by the anti-cathepsin L antibody staining, the probe also matched cathepsin L localization, which clearly demonstrated that these two enzymes share the same endolysosomal compartments, although we also detected red spots that lacked cathepsin L (Fig. 6B). The analysis of other cell lines showed a major overlap between the MP-CB-2 probe and the anti-cathepsin B antibody (Fig. S9B–S14B†), and revealed that in some cells cathepsin B and L shared the same localization (U-2 OS, HCT-166), while in others the distribution of cathepsin B and L was uneven (SK-OV-3, PC-3, OVCAR-5, MDA-MB-231). Importantly, in all tested cell lines, cathepsin B labeling by the probe was prevented when cells were preincubated with E-64d inhibitor (Fig. S15†) or when pretreated with cathepsin B cell-permeable CA-074Me inhibitor (A431 and SK-OV-3 cells, Fig. S16†). However, we must emphasize here, that although CA-074Me is generally considered as cathepsin B selective inhibitor (when intracellularly converted into methyl-free CA-074 analogue), it might still display some cross-reactivity with other cathepsins.^49^ To get a better insight in the MP-CB-2/cathepsin-B and MP-CB-2/cathepsin-L distribution in A-431 cells, we analyzed randomly selected areas from the confocal images and created Alexa488/Cy5/DAPI signal intensity plots (Fig. 6C). A detailed analysis of the distribution of fluorescence pixels from the entire images enabled us to calculate weighted colocalization coefficient (wcc),^43^ which holds the information about the colocalization between ABP and cathepsin B and between ABP and cathepsin L (Fig. 6D). In all seven cell lines tested we noticed high spatial correlation between MP-CB-2 probe and cathepsin B antibody (wcc ranging from 0.916 in PC-3 cells to 0.984 in HCT-116 cells), and significantly lower correlation between this ABP and cathepsin L antibody (from 0.682 in PC-3 cells to 0.937 in HCT-116 cells) (Table S4†). Since MP-CB-2 probe does not react with cathepsin L, the correlation between cathepsin B probe and cathepsin L antibody demonstrates that in most cancer cells cathepsin B and cathepsin L reside in the same lysosomes/endosomes, as indicated by high wcc. However there are also some cells (*i.e.* PC-3 and MDA-MB-231) where the differences in the distribution between cathepsin B and cathepsin L are more pronounced (wcc = 0.682 and 0.694, respectively). Interestingly, our data also demonstrate that red (probe) and green (antibody) signals could overlap with nuclei staining, which indicated the presence of active cathepsin B in the nuclei of A-431 cells. This data is in agreement with previously published studies that demonstrated nuclear localization of cathepsin B in these cells.^50^

### 3.7. Labeling of the membrane-bound cathepsin B

**Fig. 7:**
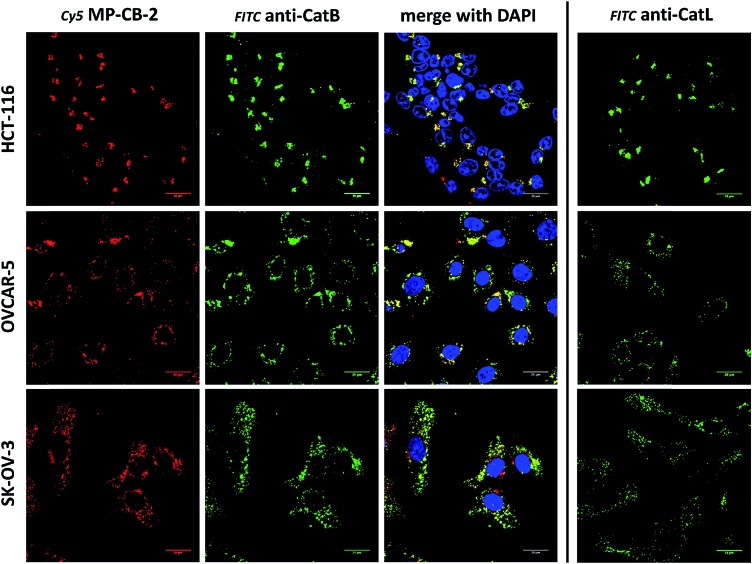
Cathepsin B detection in lysosomes of cancer cells. Cy5 MP-CB-2 probe (red) labels active cathepsin B in the lysosomes of three cancer cell lines (HCT-116, OVCAR-5 and SK-OV-3). The application of anti-cathepsin B antibody (green) demonstrates high degree of ABP selectivity. Staining cells with anti-cathepsin L antibody shows very similar distribution of this enzyme in tested cancer cells (right panel).

**Fig. 8:**
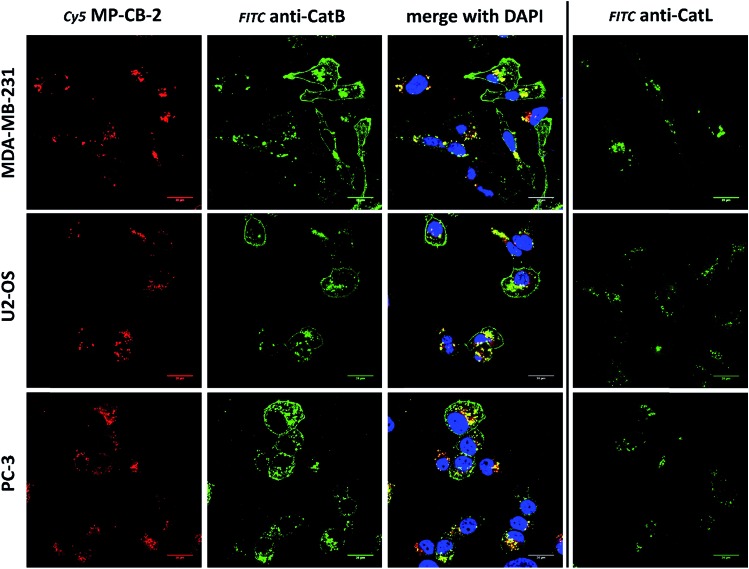
Cathepsin B detection in membranes and lysosomes of cancer cells. Application of anti-cathepsin B antibody (green) demonstrates the lysosomal and membrane-bound cathepsin B localization in MDA-MB-231, U2-OS and PC-3 cancer cells. However, MP-CB-2 probe (red) labels cathepsin B only in lysosomes. Staining cells with anti-cathepsin L antibody shows distinct cathepsin L localization, as this enzyme is present only in the lysosomes, but not in the membrane.

Cathepsin B activity has long been associated with cancer cells invasion and metastasis.^22^ In brief, cathepsin B can associate with the cell membrane through binding to annexin II tetramers, thus this association may protect cathepsin B from the unfavorable environment, and on the other hand it also can ensure that cathepsin B activity is retained on the secretion site(51). In 2012, Withana and coworkers labeled membrane-bound active cathepsin B in 3D model of 4T1.2 mammary cell line with the pan-cathepsin GB123 fluorescent ABP, demonstrating the role of this enzyme in breast cancer derived bone metastasis.^52^ Given this, we decided to use our MP-CB-2 probe in a similar context. Our immunostaining of selected cancer cells demonstrated that in some cell lines cathepsin B was found only in the endolysosomal compartments (HCT-116, OVCAR-5, SK-OV-5) (Fig. 7), whereas in the others cathepsin B was also found on the cell surface (MDA-MB-231, U2-OS, PC-3) (Fig. 8), in agreement with previous results, where cathepsin B was found to associate with the cell membrane through binding to annexin II.^51^ Our initial experiments on the above cell lines did not result in the labeling of membrane-bound cathepsin B with the ABP (Fig. 8). In order to get a better insight into this phenomenon we also tried to incubate MP-CB-2 ABP with MDA-MB-231 cells under different conditions including various pH (5.7 and 7.5) and DTT concentration (0–2.0 mM). However, regardless of conditions tested, we did not succeed in the labeling of membrane cathepsin B (Fig. S17 and S18†). We only observed that cathepsin B labeling was more intense at pH 7.5, and the addition of DTT reducing agent seemed to have no effect on this labeling. We postulate, that the lack of membrane-bound cathepsin B labeling may be due to the enzyme inactivation, alteration in specificity or limited access of the probe to the membrane compartment, as it is known that in some cell types (for instance in HCT116) cathepsin B localizes to plasma membrane caveolae.^53^,^54^ In contrast to cathepsin B, cathepsin L was present only in the endolysosomal compartments, and no membrane-bound cathepsin L was detected in any of cancer cells we tested (Fig. 7 and 8). These differences in localization of both cathepsins support their different roles in tumor invasion and metastasis.^12^

### 3.8. Labeling of cathepsin B in lung cancer patient's samples

**Fig. 9:**
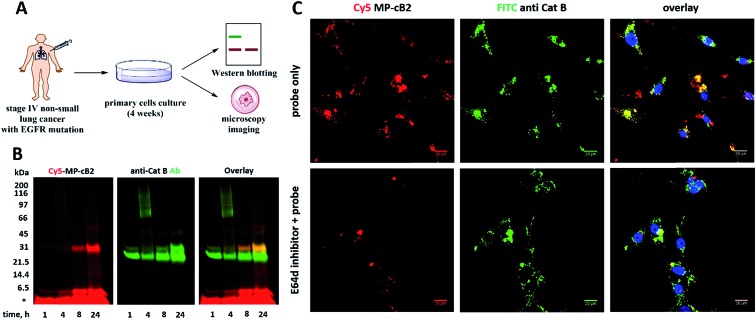
Labeling of cathepsin B in non-small lung cancer sample. (Panel A) The workflow of the experiment. Cells were collected from a cancer patient and cultured on a Petri dish. After 4 weeks cells were harvested, counted and used for experiments. (Panel B) Cathepsin B was selectively labeled in cancer cells using 1 μM MP-CB-2 ABP (left panel). This probe labeled single chain cathepsin B form (∼29 kDa) and also the light chain (∼6 kDa), which dissociated from the heavy chain after sample boiling. (Panel C) Localization of cathepsin B in cells using 1 μM of ABP (overnight incubation) and anti-cathepsin B antibody. ABP and catB antibody greatly overlapped, which demonstrates the ABP selectivity. When cells were pretreated with E64d inhibitor, the cathepsin B staining was significantly reduced.

Since the MP-CB-2 demonstrates the broad applicability for the detection of active cathepsin B across various cancer cell lines, we decided to challenge this ABP towards clinical cancer samples. For this purpose we selected non-small lung cancer, as in this disease cathepsin B is recognized as a significant prognostic marker which is directly related to anticancer therapy outcome.^55^,^56^ To test whether the MP-CB-2 probe retains the potency and selectivity in patient's sample, we collected cells from stage IV non-small lung cancer patient, cultured the cells for 4 weeks in order to collect more cells, labeled the samples with MP-cB2 ABP and subjected them for Western blotting and fluorescence microscopy analysis (Fig. 9A). Western blotting confirmed that the probe enters cancer cells and labels single (∼29 kDa) and light (∼6 kDa) chains of cathepsin B, as the signal was visible after 1 hour. The labeling was much more pronounced after 24 h, but more importantly after prolonged incubation, the probe retained selectivity (Fig. 9B). As expected, the analysis showed a major overlap between the MP-CB-2 probe and the anti-cathepsin B antibody. In a follow-up experiment, cathepsin B was also visualized using confocal fluorescence microscopy (Fig. 9C). This experiment demonstrated that MP-CB-2 probe shows lysosomal-like pattern of cathepsin B labeling, which overlaps with anti-cathepsin B antibody. We also detected some off labeling which is probably due to insufficient specimen washing. When the cells were incubated with E64d inhibitor prior to MP-CB-2 probe, the lysosomal-like cathepsin B staining was significantly reduced, however, the off-labeling was still visible. By this we demonstrated that although, the MP-CB-2 is selective and efficiently labels cathepsin B, our technical procedure for sample preparations needs to be improved in order reduce off-labeling.

## 4. Conclusions

Sequence and structure homology, overlapping functions and subcellular localization, and similar substrate specificities, have made it very difficult to discriminate between individual cysteine cathepsins and to devise reagents to track their activity *in* or *ex vivo*. In this work we applied chemical-based HyCoSuL approach for in-depth profiling of cathepsin B substrate preferences in the P4–P1 positions. The use of a wide range of unnatural amino acids in fluorescence peptide libraries, enabled us to extensively explore the unique chemical moieties of the cathepsin B-active site. We used the obtained specificity map to develop new, selective cathepsin B substrates that were not hydrolyzed by other cathepsins (L, V, S and K). All designed selective substrates shared structural similarities, such as aliphatic amino acids in the P4 and P3 positions, bulky and long aromatic amino acids in the P2 position, and Arg in the P1 position. The best cathepsin B substrate (Ac-Cha-Leu-Glu(Bzl)-Arg-ACC) was ∼3500-fold selective for cathepsin B when compared in comparison with cathepsin L, and ∼60 000-fold more selective in comparison with cathepsin S. Next, we utilized the most selective peptide sequence to synthesize fluorescent cathepsin B-selective ABPs. We further demonstrated that one of such probes (MP-CB-2) was taken up by cancer cells and could selectively label cathepsin B even at prolonged incubation times. To show the broad applicability of this probe, we screened eighteen cancer cell lines with different expression levels of cathepsins. For example, in A-431 cells which displayed low cathepsin B and high cathepsins L and S expression, MP-CB-2 probe labeled only cathepsin B even after 24 hours of incubation. As expected, the MP-CB-2 cellular uptake differed across the different cell lines, as in some cell lines cathepsin B was labeled after 1 hour, whereas in the others the labeling was detectable only after 8 hours. We also performed fluorescence microscopy analysis and used the MP-CB-2 probe to localize active cathepsin B in cells. Although MP-CB-2 displays almost negligible cross-reactivity, we showed that the probe signal and cathepsin L antibody staining greatly overlapped in tested cell lines, suggesting that cathepsins B and L share the same cellular localization. Notably, in some cell lines (MDA-MB-231, U2-OS, and PC-3) cathepsin B, but not cathepsin L, was found bound to the membrane, which support their different functions in cancer. However, no cell surface cathepsin B activity was detected with the MP-CB-2 probe, which needs to be further explored. Finally, we used our ABP to label cathepsin B in non-small lung cancer cells collected from oncological patient. The Western blotting and fluorescence microscopy analyses showed selective cathepsin B detection, demonstrating that MP-CB-2 is also useful in clinical settings. The ability of MP-CB-2 probe to selectively detect cathepsin B in patient samples is of great importance, as this enzyme very often serves as prognostic marker for multiple cancer types. In summary, we developed a highly selective cathepsin B ABP that allows accurate detection of cathepsin B activity in various cancer cells, regardless of the cathepsin B expression level, and the activities of other cathepsins. Moreover, this ABP can also serve as a cathepsin B-selective inhibitor that may be useful in further studies of biological functions of this enzyme.

## Author contributions

M. P., M. D., and G. S. S. designed research; M. P. performed research and collected data; K. G. synthesized activity-based probes and performed all experiments with cancer patient's samples, M. V., D. T., and B. T. contributed new reagents and enzymes; M. M. and G. P. contributed patient's cancer cells and helped to design and perform experiments; M. P., M. D., and G. S. S. analyzed and interpreted the data; M. P. wrote the draft of the manuscript and K. G., M. V., M. M., D. T, B. T., G. P., M. D., and G. S. S. critically revised and edited the manuscript.

## Conflicts of interest

There are no conflicts to declare.

## Supplementary Material

Supplementary information
