# Supplementary material for: Fluorescent probes towards selective cathepsin B detection and visualization in cancer cells and patient samples
Source: Chem Sci. 2019 Jul 31;10(36):8461–77. doi: 10.1039/c9sc00997c (PMC6839509; doi:10.1039/c9sc00997c)
Supplement: Supplementary file 1 [file SC-010-C9SC00997C-s001.pdf]

Electronic Supplementary Material (ESI) for Chemical Science.
This journal is © The Royal Society of Chemistry 2019

Fluorescent probes towards selective cathepsin B detection and visualization in cancer cells and patient samples

Marcin Poreba[1,2], Katarzyna Groborz[2], Matej Vizovisek[3], Marco Maruggi[1], Dusan Turk[3], Boris Turk[3,4], Garth Powis[1], Marcin Drag[1,2], Guy S. Salvesen[1]

[1]Sanford Burnham Prebys Medical Discovery Institute, 10901 North Torrey Pines Road, La Jolla, CA 92037, USA

[2]Department of Bioorganic Chemistry, Faculty of Chemistry, Wroclaw University of Technology, Wyb. Wyspianskiego 27, 50-370 Wroclaw, Poland

[3]Department of Biochemistry and Molecular and Structural Biology, Jožef Stefan Institute, SI-1000 Ljubljana, Slovenia

[4]Faculty of Chemistry and Chemical Technology, University of Ljubljana, SI-1000 Ljubljana, Slovenia

Corresponding authors: marcin.poreba@pwr.edu.pl, marcin.drag@pwr.edu.pl, gsalvesen@sbpdiscovery.org,

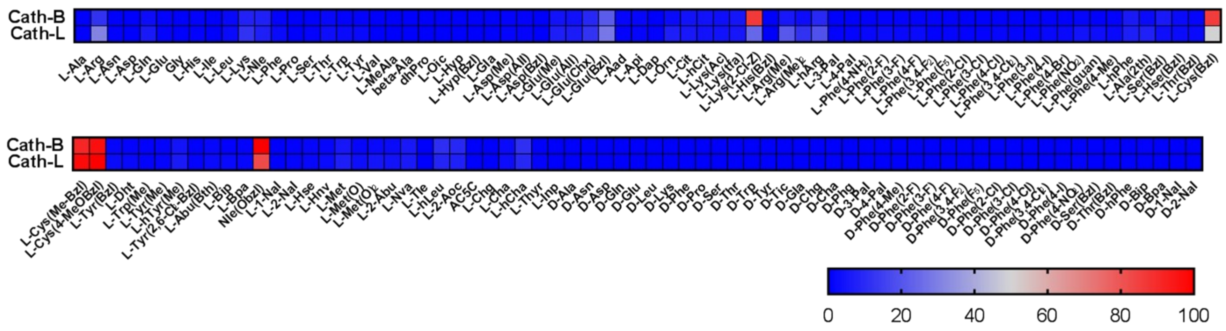

Figure 1 P1 substrate specificity of cathepsin L and B in the P1 position. Cathepsin B and cathepsin L specificity in the P1 position was determined using Ac-Ala-Arg-Leu-P1-ACC individual substrate library and presented as heat-maps. The value for the best recognized amino acid was set as 100 % (red) and other amino acids were adjusted accordingly (blue/red scale). Cathepsin L P1 substrate specificity was adopted from our previous study (Poreba et al., Chemical Science, 2018).

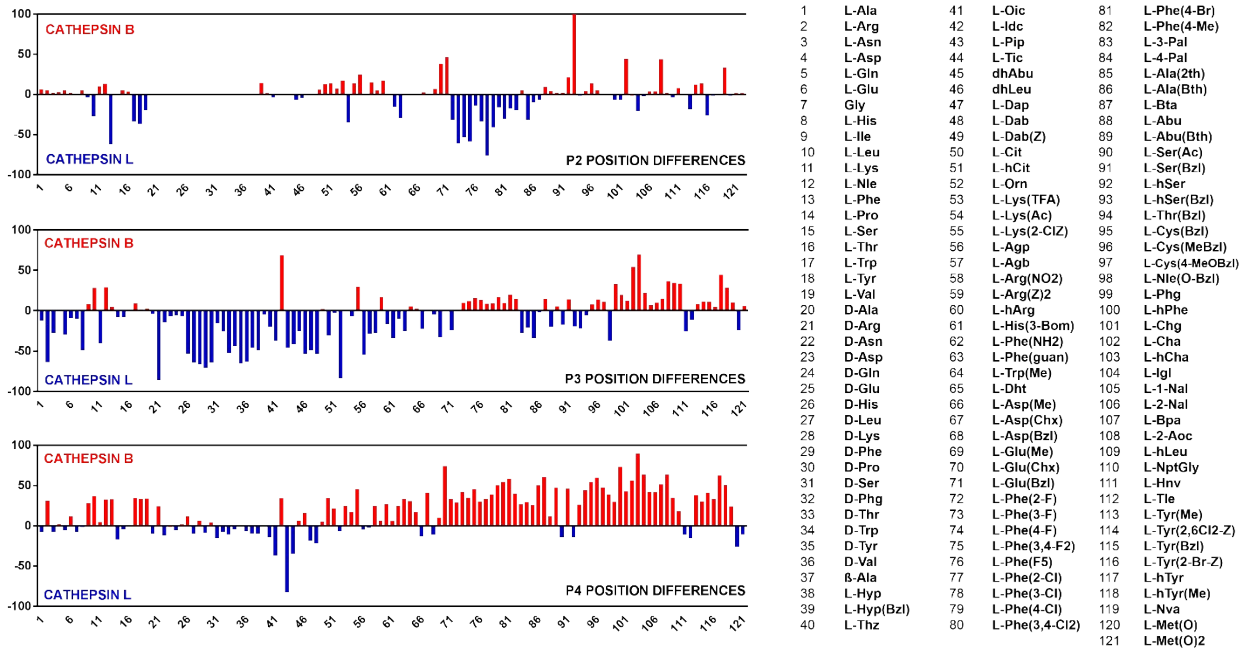

Figure 2 Differences in P4-P2 substrate specificities between cathepsin B and cathepsin L. Substrate specificity of cathepsin L (Poreba et al. Chemical Science 2018) and cathepsin B in P4-P2 positions were profiled using HyCoSuL approach. Here we present the differences between these enzymes, calculated for each amino acid using the formula: catB activity (%) – catL activity (%). Red bars indicate amino acids that were preferred by cathepsin B over cathepsin L (y axis; 0-100 %). Blue bars indicate amino acids that were preferred by cathepsin L over cathepsin B (y axis; -100 – 0 %). Amino acids without bars (y axis = 0)

were tolerated by both enzymes at the same level or were not recognized at all. The x axis shows the number of amino acids in the HyCoSuL library, and the amino acids abbreviation is shown in the right panel.

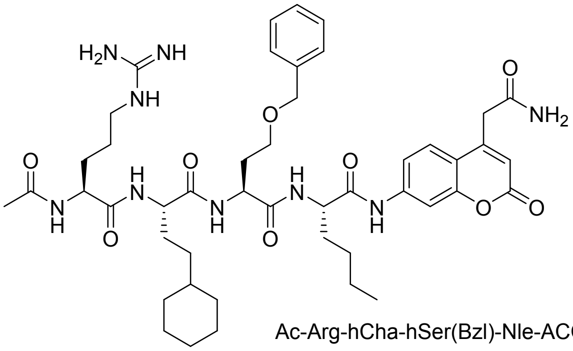

Ac-Arg-hCha-hSer(Bzl)-Nle-ACC

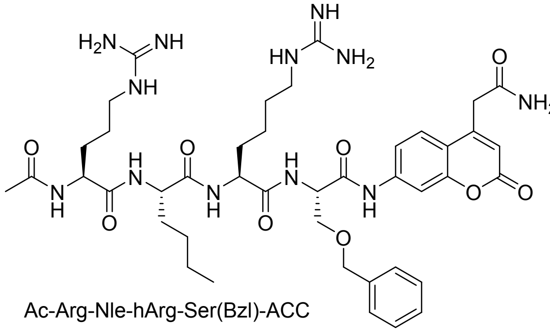

Ac-Arg-Nle-hArg-Ser(Bzl)-ACC

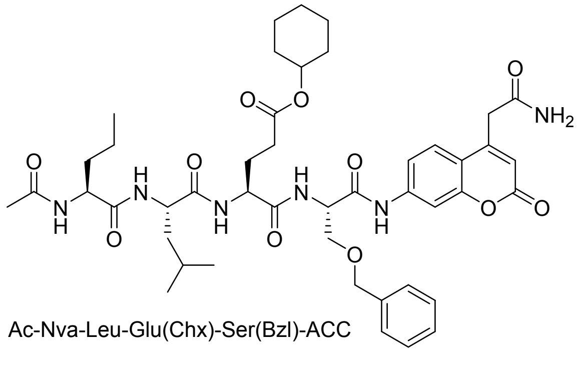

Ac-Nva-Leu-Glu(Chx)-Ser(Bzl)-ACC

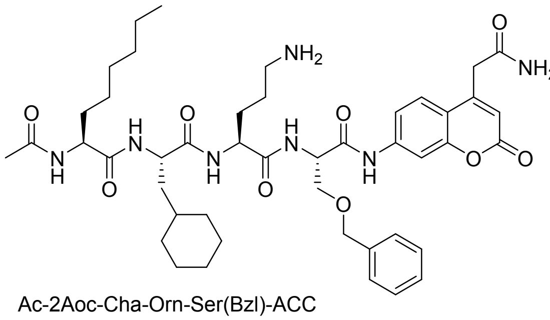

Ac-2Aoc-Cha-Orn-Ser(Bzl)-ACC

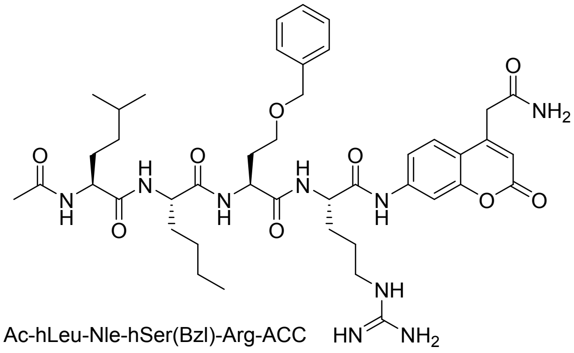

Ac-hLeu-Nle-hSer(Bzl)-Arg-ACC

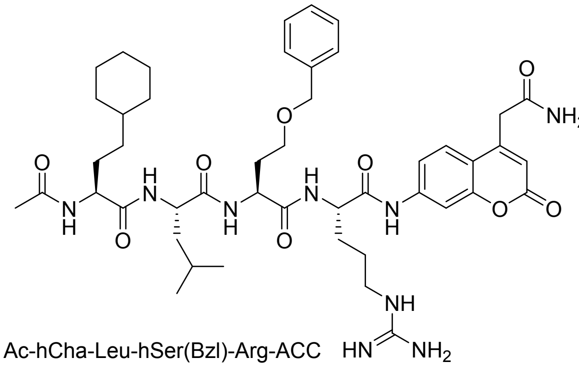

Ac-hCha-Leu-hSer(Bzl)-Arg-ACC

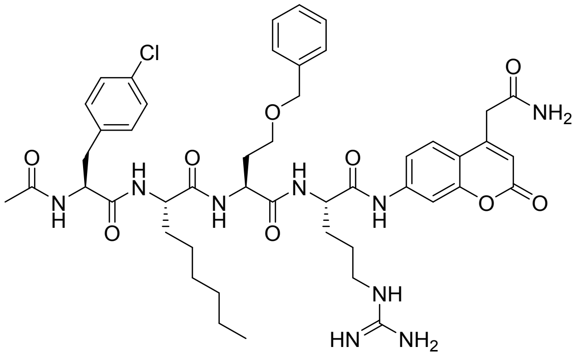

Ac-Phe(4Cl)-2Aoc-hSer(Bzl)-Arg-ACC

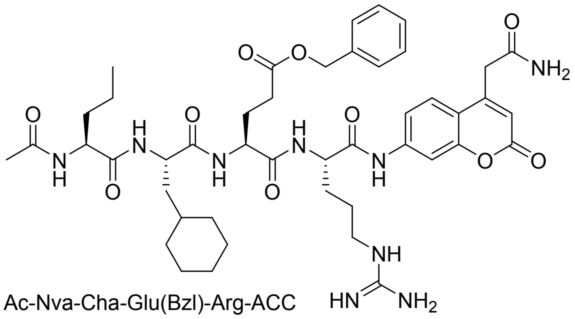

Ac-Nva-Cha-Glu(Bzl)-Arg-ACC

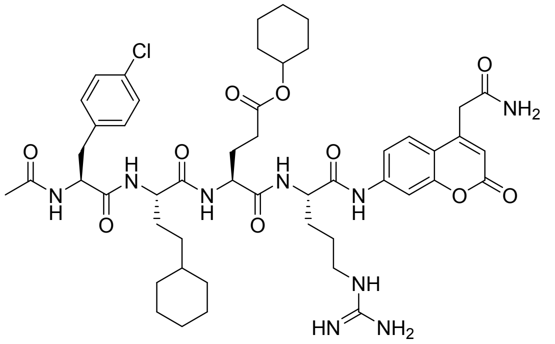

Ac-Phe(4Cl)-hCha-Glu(Chx)-Arg-ACC

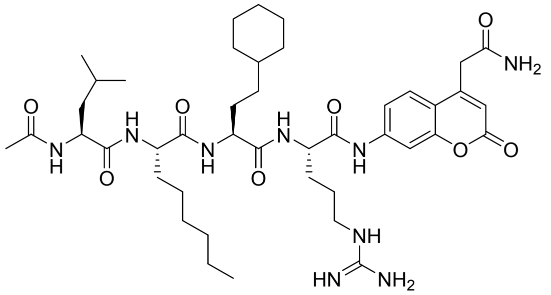

Ac-Leu-2Aoc-hCha-Arg-ACC

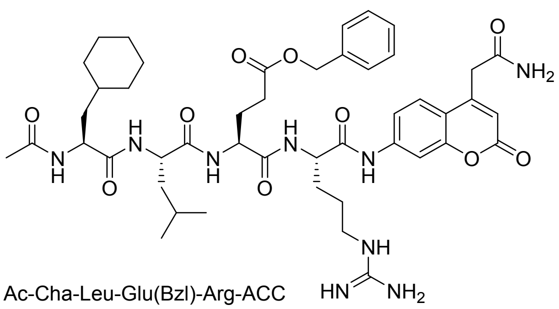

Ac-Cha-Leu-Glu(Bzl)-Arg-ACC

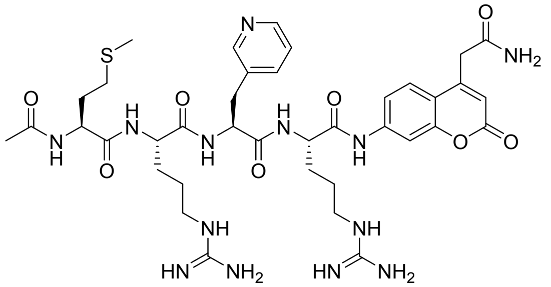

Ac-Met-Arg-3Pal-Arg-ACC

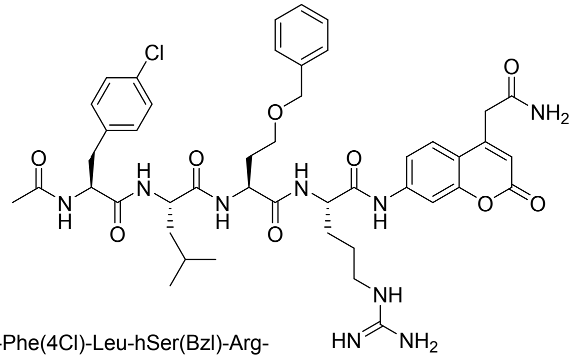

Ac-Phe(4Cl)-Leu-hSer(Bzl)-Arg-ACC

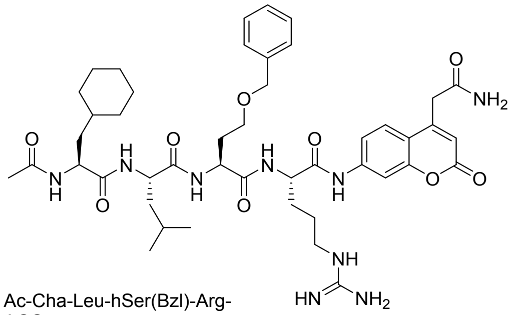

Ac-Cha-Leu-hSer(Bzl)-Arg-ACC

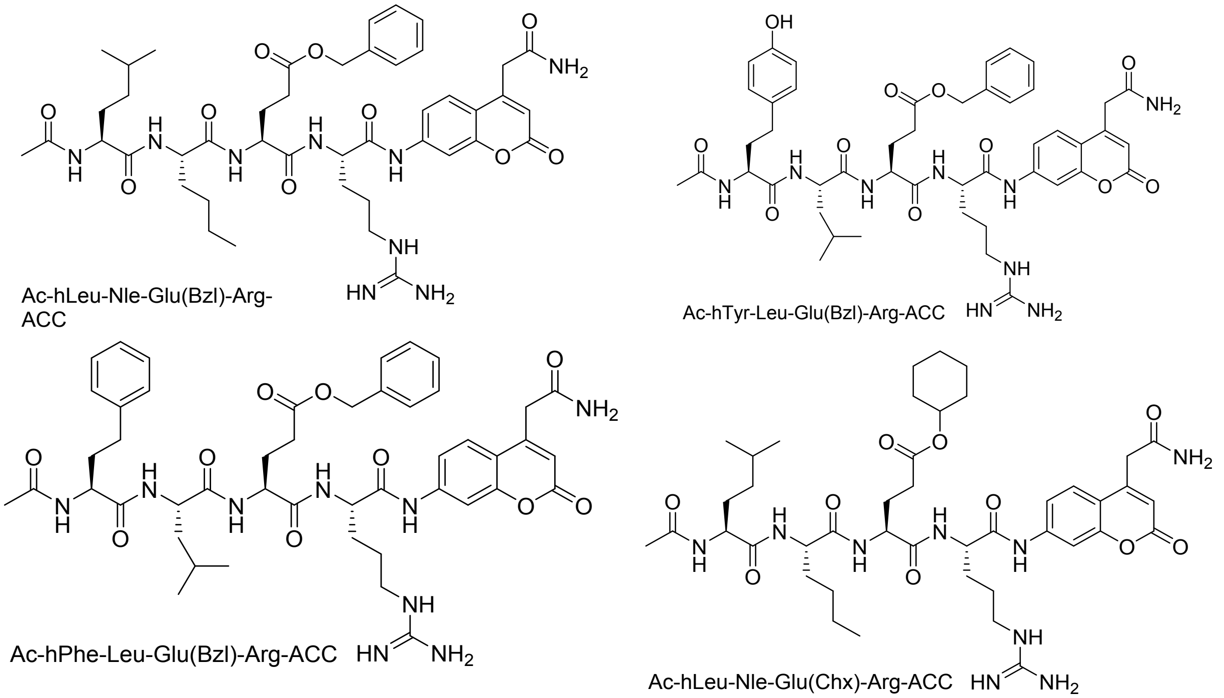

Table 1 Structures of ACC-labeled tetrapeptide substrates selective for cathepsin B.

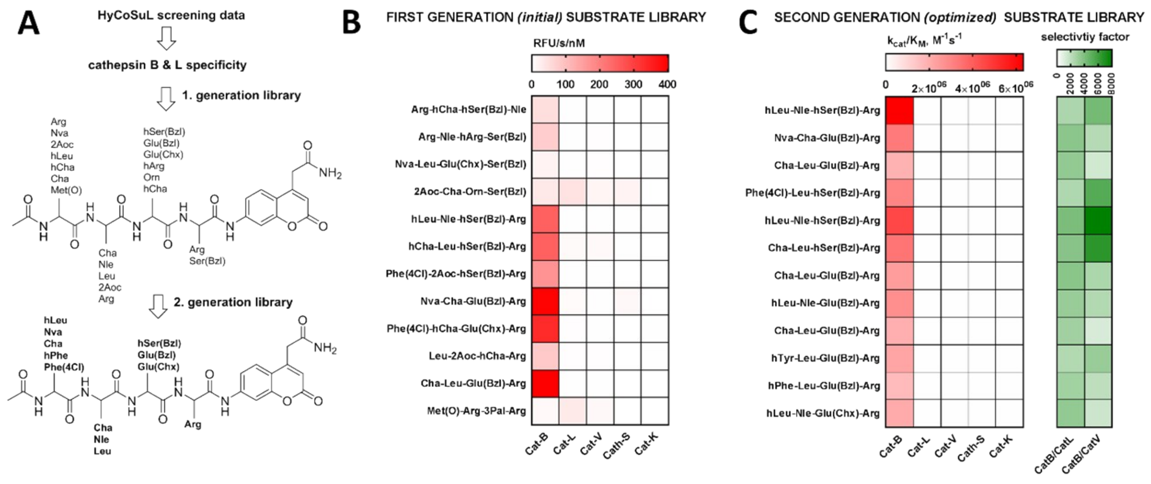

Figure 3 The procedure for the optimization of cathepsin B selective substrate. Panel A. Cathepsin B and cathepsin L substrate preferences were determined using HyCoSuL approach. Next, a 12-membered library of potentially cathepsin B selective substrates were synthesized and kinetically evaluated towards five recombinant cathepsins (B, L, V, S, and K). Next, an optimized set of cathepsin B selective substrates was synthesized and evaluated. **Panel B.** The hydrolysis rates (RFU/s/nM) of 1st generation of cathepsin B substrates (2 μM) by five recombinant cathepsins (5 nM). **Panel C.** The kinetic parameters of substrate hydrolysis (k_{cat}/K_M) for the 2nd generation of cathepsin B substrates were measured for five recombinant cathepsins (red panel) and the substrate selectivity factors of selected cathepsin B substrates were calculated for cathepsins L and V (green panel). The selectivity of all 2nd generation cathepsin B substrates over cathepsin S and K were over 30,000-fold.

Ac-peptide-ACC	Cat B	Cat L	Cat V	Cat S	Cat K
			k_{cat}/K_M, $M^{-1}s^{-1}$		
hLeu-Nle-hSer(Bzl)-Arg	7,900,000	3,130	1,460	65	19
Nva-Cha-Glu(Bzl)-Arg	4,090,000	1,140	1,420	37	30
Phe(4Cl)-Leu-hSer(Bzl)-Arg	3,780,000	1,540	570	50	12
hLeu-Nle-hSer(Bzl)-Arg	5,700,000	1,390	577	39	16
Cha-Leu-hSer(Bzl)-Arg	4,300,000	1,040	518	44	14
Cha-Leu-Glu(Bzl)-Arg	3,100,000	744	949	40	14
hLeu-Nle-Glu(Bzl)-Arg	3,450,000	1,090	1,180	96	17
hTyr-Leu-Glu(Bzl)-Arg	2,760,000	1,170	692	41	14
hPhe-Leu-Glu(Bzl)-Arg	2,100,000	726	829	37	12
hLeu-Nle-Glu(Chx)-Arg	2,600,000	769	1,310	25	11

Table 2 Kinetic parameters for the substrate hydrolysis (k_{cat}/K_M) of the 2nd generation cathepsin B substrates measured towards five recombinant cathepsins.

	k_{cat}/K_M, $M^{-1}s^{-1}$	K_M, μM	k_{cat}, s^{-1}
	Substrate: **Ac-Cha-Leu-hSer(Bzl)-Arg-ACC**		
Cat B	4,300,000	90.9	390
Cat L	1,040	105	0.109
Cat V	518	109	0.055
Cat S	44	> 500	< 0.022
Cat K	14	> 500	< 0.007
	Substrate: **Ac-Cha-Leu-Glu(Bzl)-Arg-ACC**		
Cat B	3,100,000	115	344
Cat L	744	83.5	0.062
Cat V	949	35.1	0.034
Cat S	40	> 500	< 0.020
Cat K	14	> 500	< 0.007

Table 3 Detailed kinetic parameters of substrate hydrolysis of two cathepsin B selective ACC substrates. The data demonstrate that the selectivity is mainly driven by k_{cat}, whereas K_M has almost no impact on the selectivity. All parameters were calculated from four independent experiments, and S.D. for all values are below 15%.

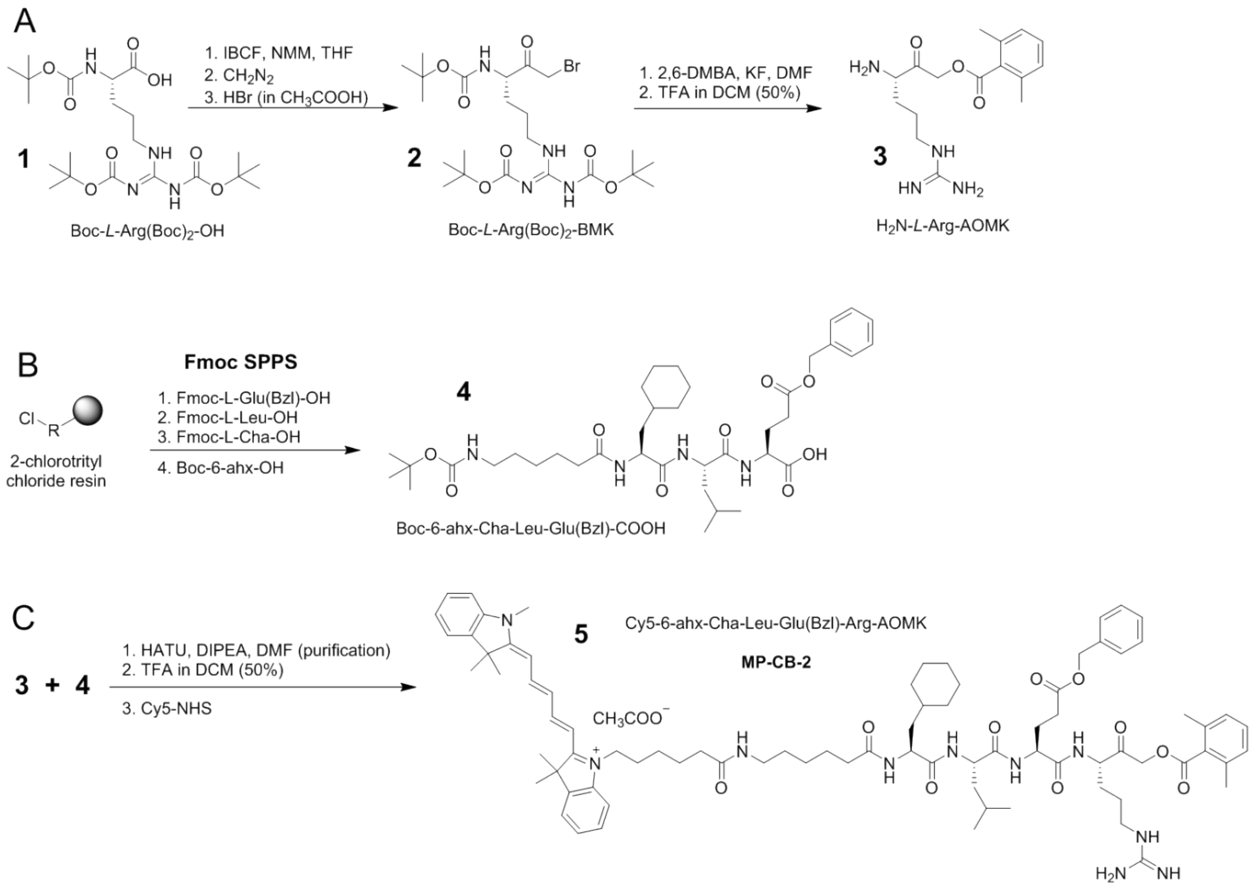

Figure 4 A schematic representation of the synthesis of MP-CB-5 activity-based probe. Abbreviations: IBCF – isobutyl chloroformate, NMM – N-methylmorpholine, THF – tetrahydrofurane, 2,6-DMBA – 2,6-dimethylbenzoic acid, SPPS – solid phase peptide synthesis, Cy5-NHS Cy5 N-hydroxysuccinimide, AOMK – 2,6-dimethylacyloxymethylketone.

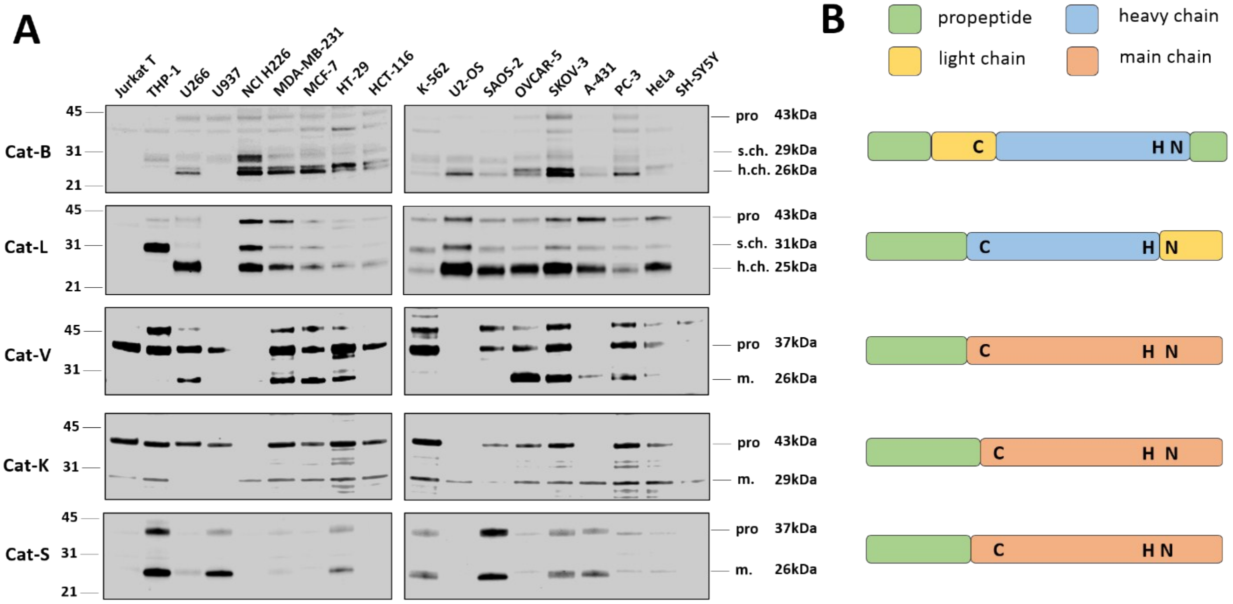

Figure 5 Detection of cysteine cathepsins in human cancer cells. Panel A Five human cathepsins (-B, -L, -V, -S, and –K) were detected in the lysates of eighteen human cancer cell lines using specific anti-human cathepsin antibodies. Equal amount of cell lysates (calculated based on total protein concentration - 13mg/mL determined at A_{280} nm with BSA standard curve, and confirmed by Instant Blue staining) were loaded on the 10 well gels followed by SDS-PAGE and Western blot analysis. For each cathepsin three/two forms were detected and assigned (pro – proenzyme, s.ch. – single chain, h.ch. heavy chain, m. – main chain). **Panel B** Schematic architecture of five human cathepsins. Cartoon adapted from Olson and Joyce, Nature Review Cancer, 2015, 15, 712.

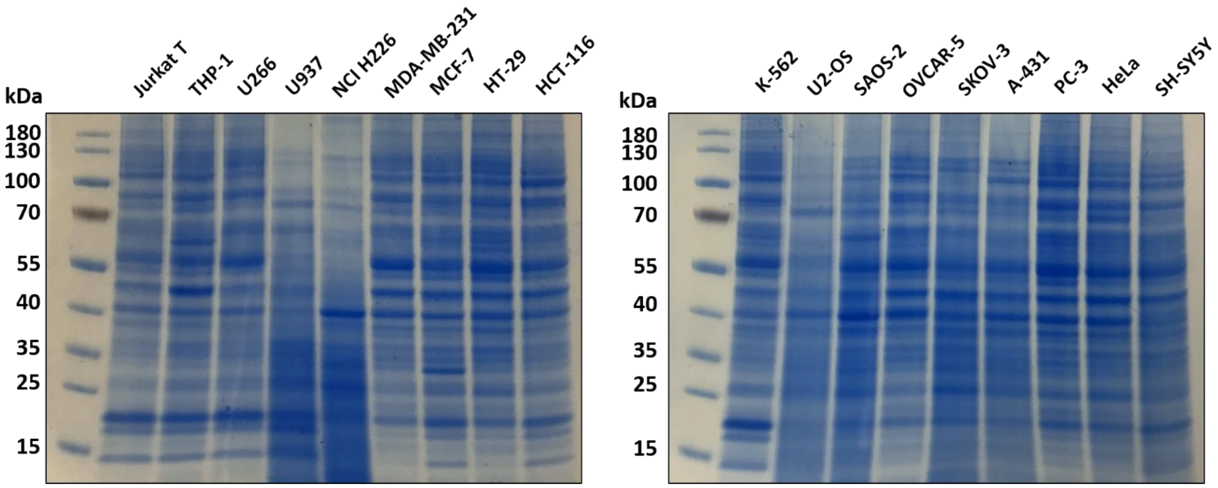

Figure 6 Gels stained with Instant Blue demonstrate equal protein loading across a panel of 18 human cancer cell lines.

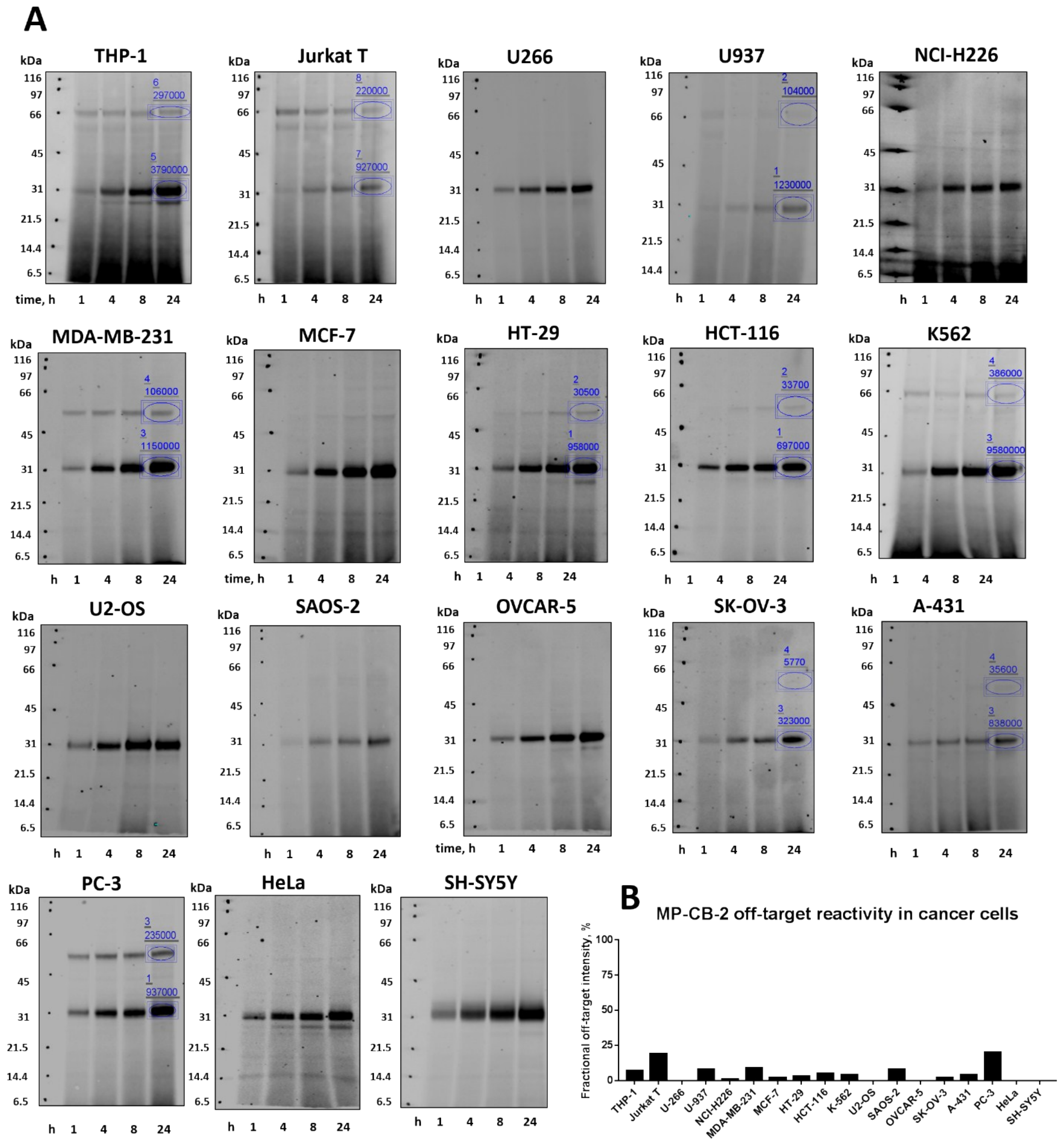

Figure 7 MP-CB-2 off-target reactivity in cancer cells. Panel A. Cathepsin-B selective probe displays some cross-reactivity with unknown, non-cathepsin protein (around 50-55 kDa) in cancer cells. To quantify the level of MP-CB-2 off-target reactivity we measured the fluorescence intensity of both bands after 24 hours (from cathepsin B and unknown protein labeling) and calculated the percentage of total labeling signal (both bands) from unknown protein labeling. Blue circles indicate the blot area taken for the analysis. **Panel B.** Analysis of signal intensities from a panel of eighteen cell lines blots demonstrates that there is a medium off-target activity in PC-3 cells (20% off-target) and Jurkat T cells (20%), and very weak to no off-target activity in other cell lines tested (below 10%).

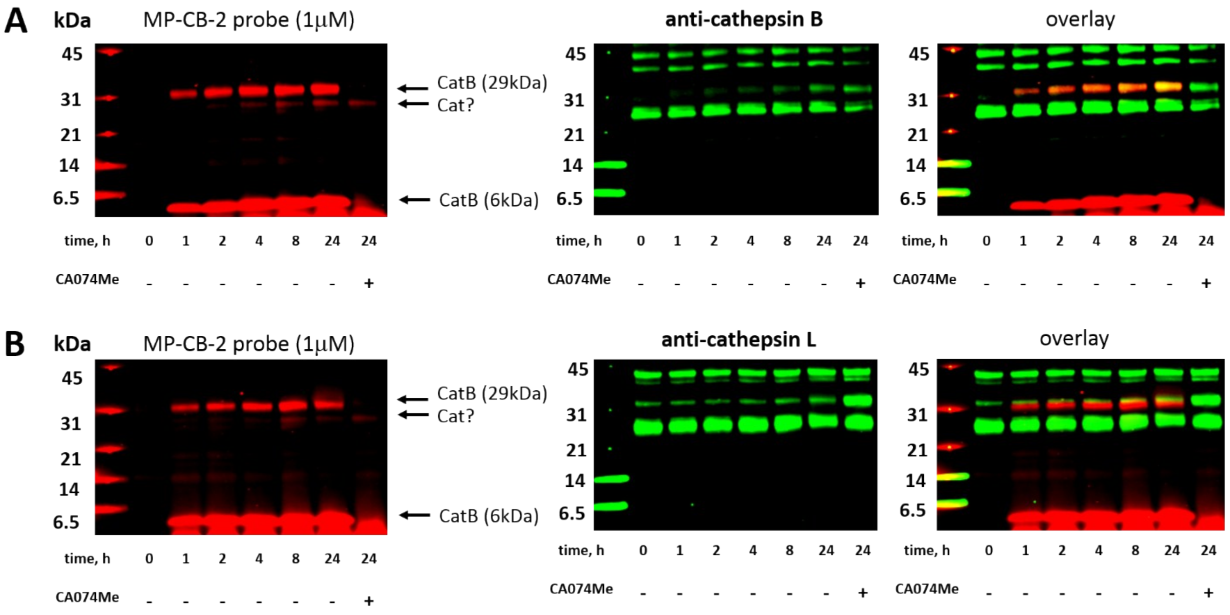

Figure 8 Cathepsin B labeling in A431 cancer cells using MP-CB-2 activity-based probe. Panel A Active cathepsin B (6 kDa and 29 kDa) was efficiently labeled in living A431 cells with 1μM MP-CB-2 probe. The use of cell-permeable, cathepsin B selective CA-074Me inhibitor resulted in no enzyme labeling. After prolonged incubation (24 hours) a slight off-target labeling was detected (27-29 kDa), which was not CA074Me-dependent. **Panel B** Cathepsin B labeling (red) correlated with anti-cathepsin L antibody (green). The analysis showed that the off-labeling (27-29 kDa) does not come from active cathepsin L (around 30 kDa).

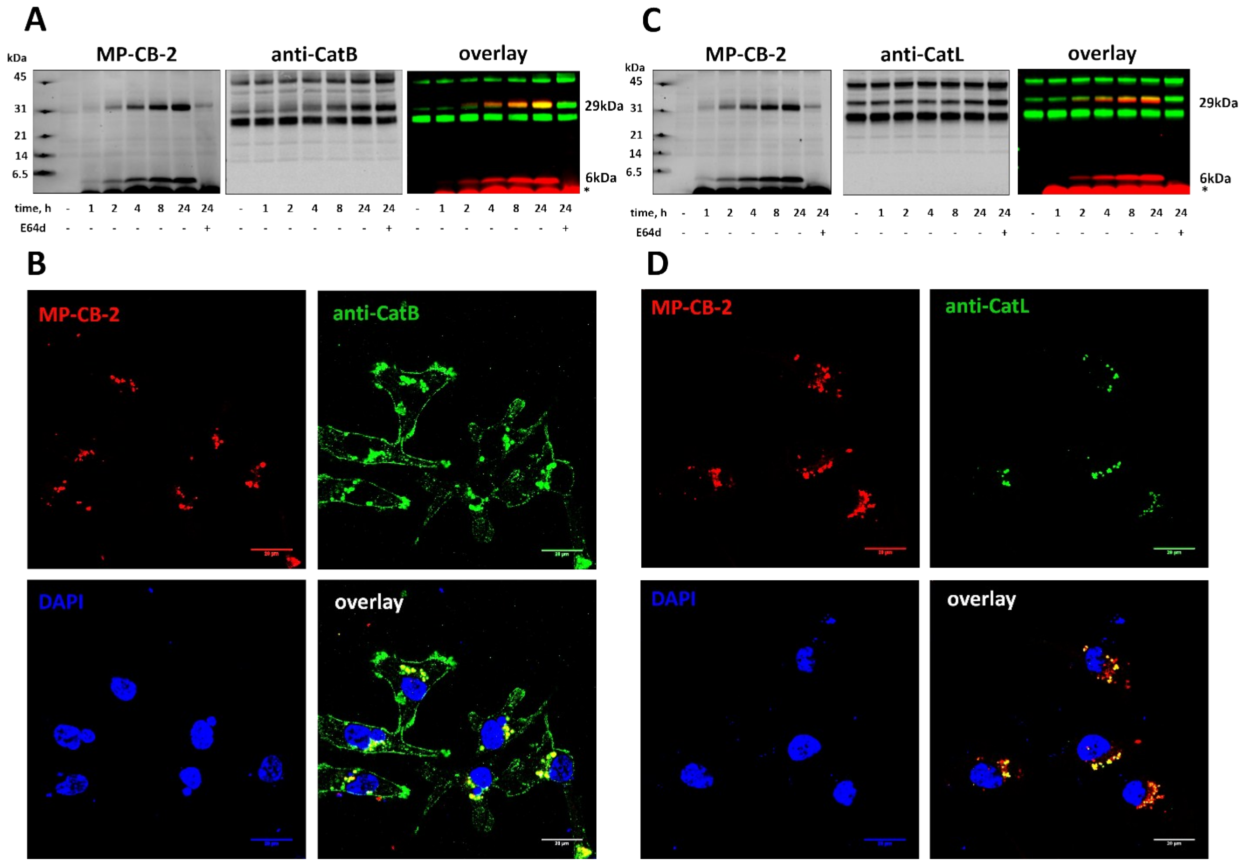

Figure 9 Cathepsin B labeling in MDA-MB-231 cancer cells using MP-CB-2 probe. Panel A and C. Cathepsin B was selectively labeled in MDA-MB-231 cell line using 1 μM MP-CB-2 ABP (Panel A). This probe labels single chain of cathepsin B form (~29 kDa) and also a light chain (~6 kDa) which is formed after sample boiling. The cathepsin B labeling can be prevent by using E-64d inhibitor. MP-CB-2 probe does not label cathepsin L even after prolonged incubation (24 hours, Panel C). Asterix (*) indicates unbound MP-CB-2 probe that migrated through the whole gel. Time point "-" indicates no probe was added. **Panel B and D.** Localization of cathepsin B in MDA-MB-231 cells using 1 μM of MP-CB-2 ABP (overnight incubation) and anti-cathepsin B antibody. MP-CB-2 and cat-B antibody greatly overlap, which confirms the high selectivity of the probe. The signal from the probe only partially overlaps with anti-cathepsin L antibody, demonstrating that both enzymes share the same subcellular localization, however there are also some cathepsin B-rich lysosomes lacking cathepsin L.

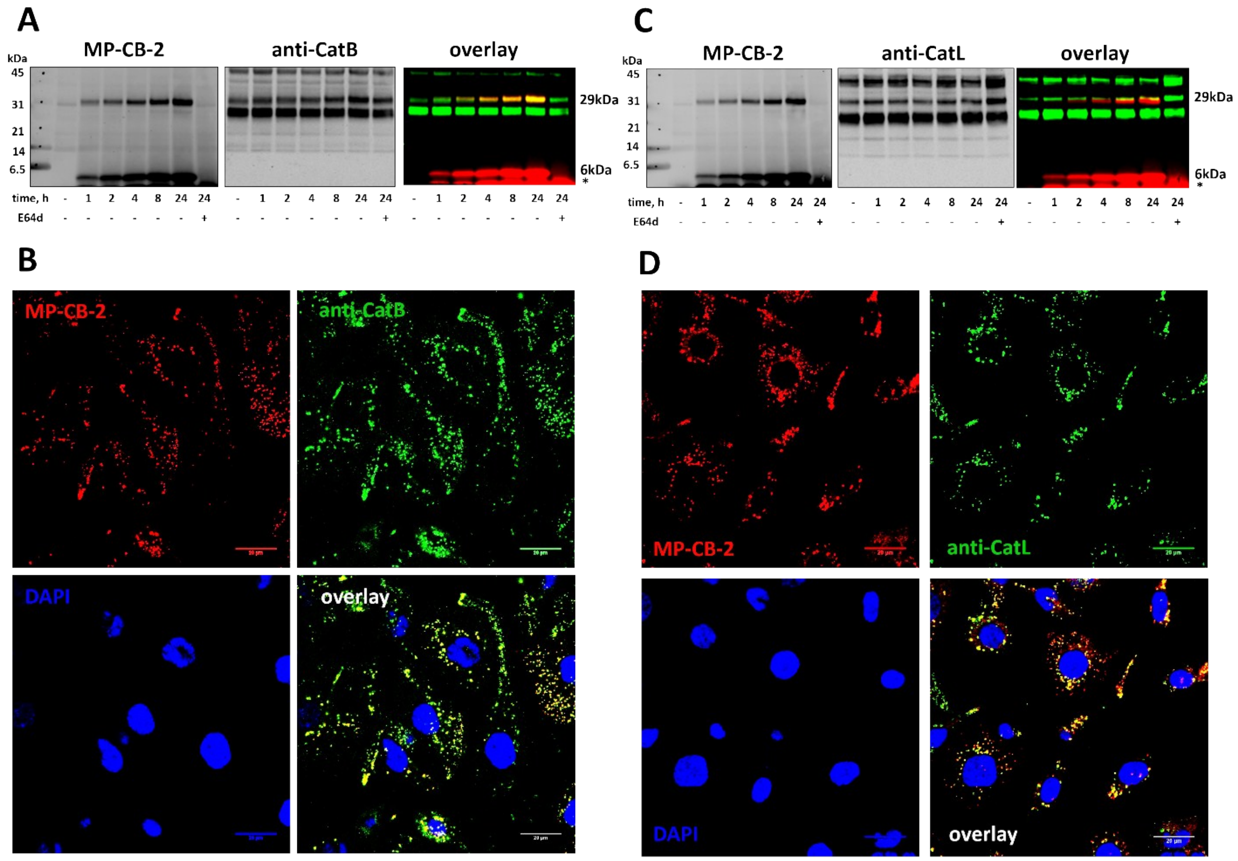

Figure 10 Cathepsin B labeling in OVCAR-5 cancer cells using MP-CB-2 probe. Panel A and C. Cathepsin B was selectively labeled in OVCAR-5 cell line using 1 µM MP-CB-2 ABP (Panel A). This probe labels single chain of cathepsin B form (~29 kDa) and also a light chain (~6 kDa) which is formed after sample boiling. The cathepsin B labeling can be prevent by using E-64d inhibitor. MP-CB-2 probe does not label cathepsin L even after prolonged incubation (24 hours, Panel C). Asterix (*) indicates unbound MP-CB-2 probe that migrated through the whole gel. Time point "-" indicates no probe was added. **Panel B and D.** Localization of cathepsin B in OVCAR-5 cells using 1 µM of MP-CB-2 ABP (overnight incubation) and anti-cathepsin B antibody. MP-CB-2 and cat-B antibody greatly overlap, which confirms the high selectivity of the probe. The signal from the probe only partially overlaps with anti-cathepsin L antibody, demonstrating that both enzymes share the same subcellular localization, however there are also some cathepsin B-rich lysosomes lacking cathepsin L

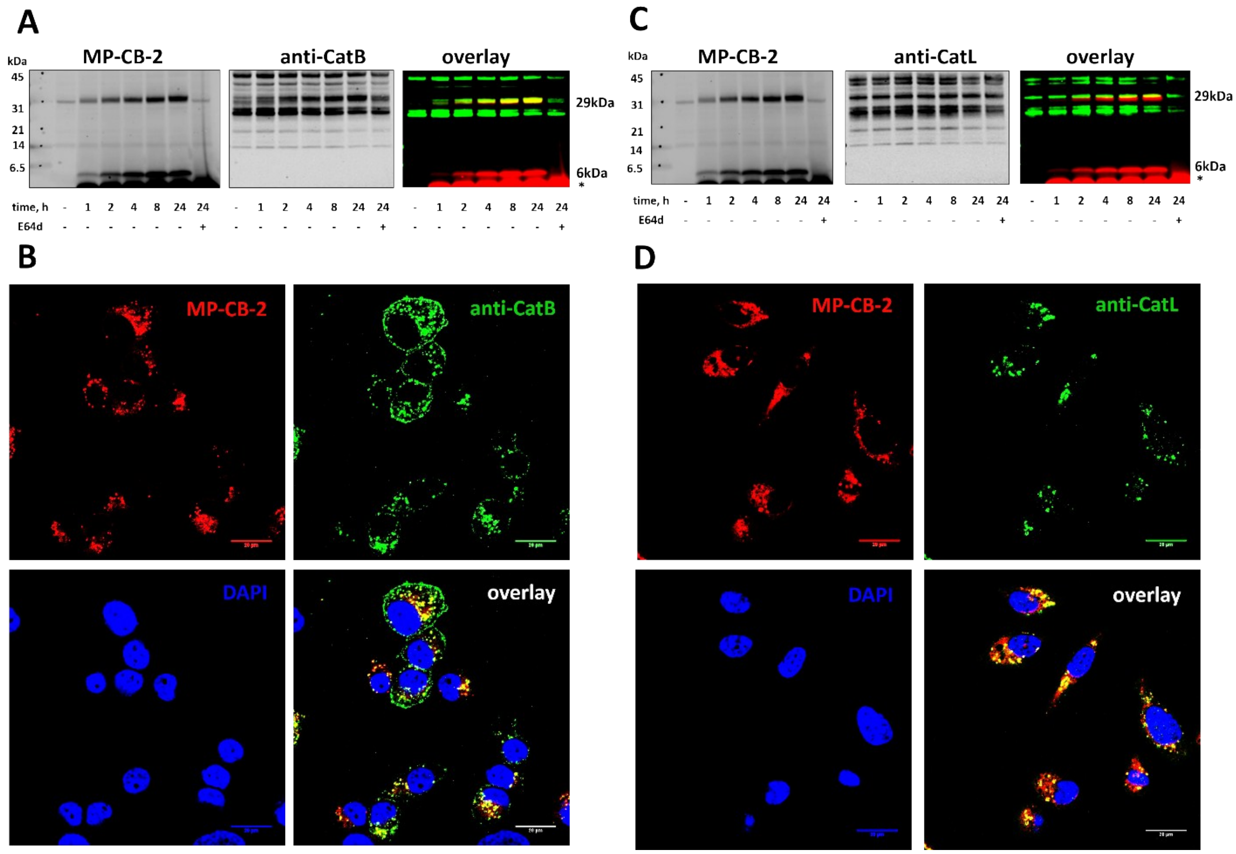

Figure 11 Cathepsin B labeling in PC-3 cancer cells using MP-CB-2 probe. Panel A and C. Cathepsin B was selectively labeled in PC-3 cell line using 1 µM MP-CB-2 ABP (Panel A). This probe labels single chain of cathepsin B form (~29 kDa) and also a light chain (~6 kDa) which is formed after sample boiling. The cathepsin B labeling can be prevent by using E-64d inhibitor. MP-CB-2 probe does not label cathepsin L even after prolonged incubation (24 hours, Panel C). Asterix (*) indicates unbound MP-CB-2 probe that migrated through the whole gel. Time point "-" indicates no probe was added. **Panel B and D.** Localization of cathepsin B in PC-3 cells using 1 µM of MP-CB-2 ABP (overnight incubation) and anti-cathepsin B antibody. MP-CB-2 and cat-B antibody greatly overlap, which confirms the high selectivity of the probe. The signal from the probe only partially overlaps with anti-cathepsin L antibody, demonstrating that both enzymes share the same subcellular localization, however there are also some cathepsin B-rich lysosomes lacking cathepsin L

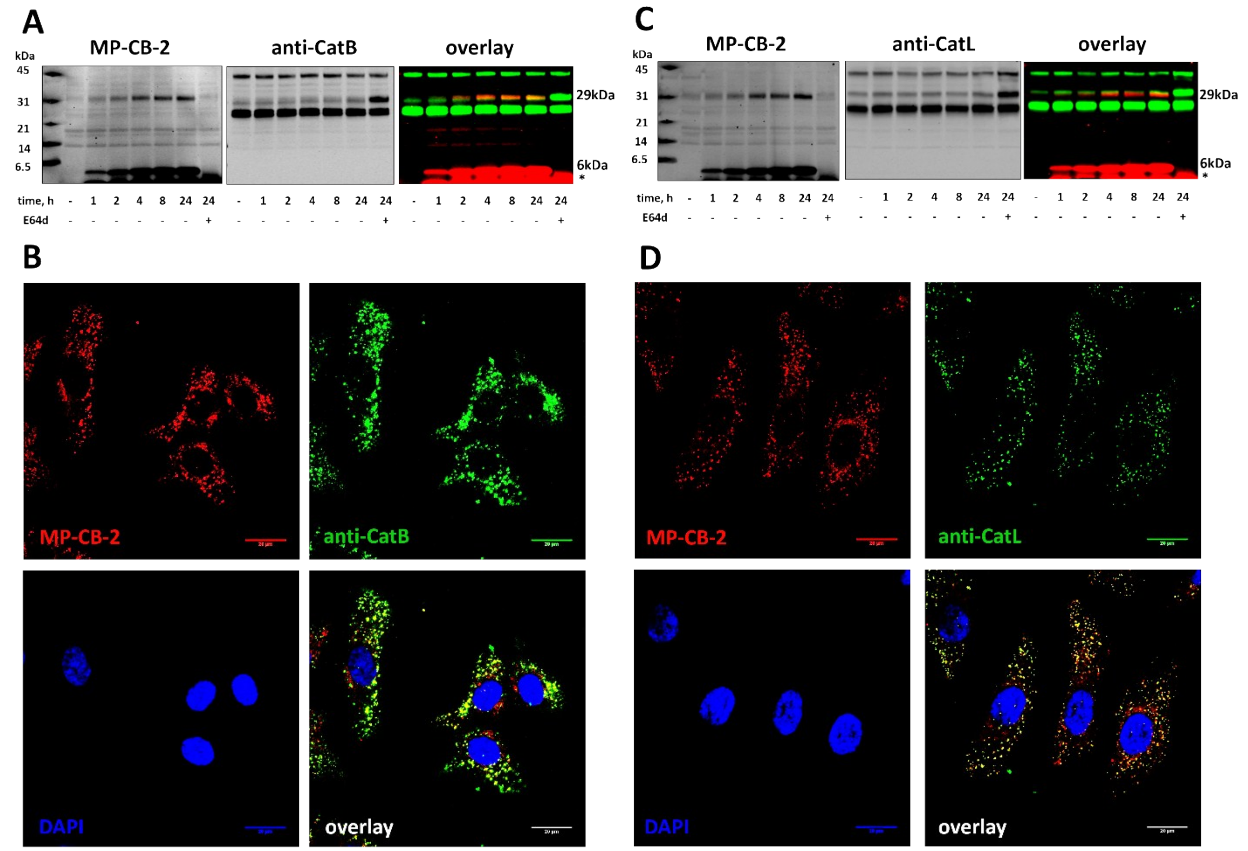

Figure 12 Cathepsin B labeling in SK-OV-3 cancer cells using MP-CB-2 probe. Panel A and C. Cathepsin B was selectively labeled in SK-OV-3 cell line using 1 μM MP-CB-2 ABP (Panel A). This probe labels single chain of cathepsin B form (~29 kDa) and also a light chain (~6 kDa) which is formed after sample boiling. The cathepsin B labeling can be prevent by using E-64d inhibitor. MP-CB-2 probe does not label cathepsin L even after prolonged incubation (24 hours, Panel C). Asterix (*) indicates unbound MP-CB-2 probe that migrated through the whole gel. Time point "-" indicates no probe was added. **Panel B and D.** Localization of cathepsin B in SK-OV-3 cells using 1 μM of MP-CB-2 ABP (overnight incubation) and anti-cathepsin B antibody. MP-CB-2 and cat-B antibody greatly overlap, which confirms the high selectivity of the probe. The signal from the probe only partially overlaps with anti-cathepsin L antibody, demonstrating that both enzymes share the same subcellular localization, however there are also some cathepsin B-rich lysosomes lacking cathepsin L

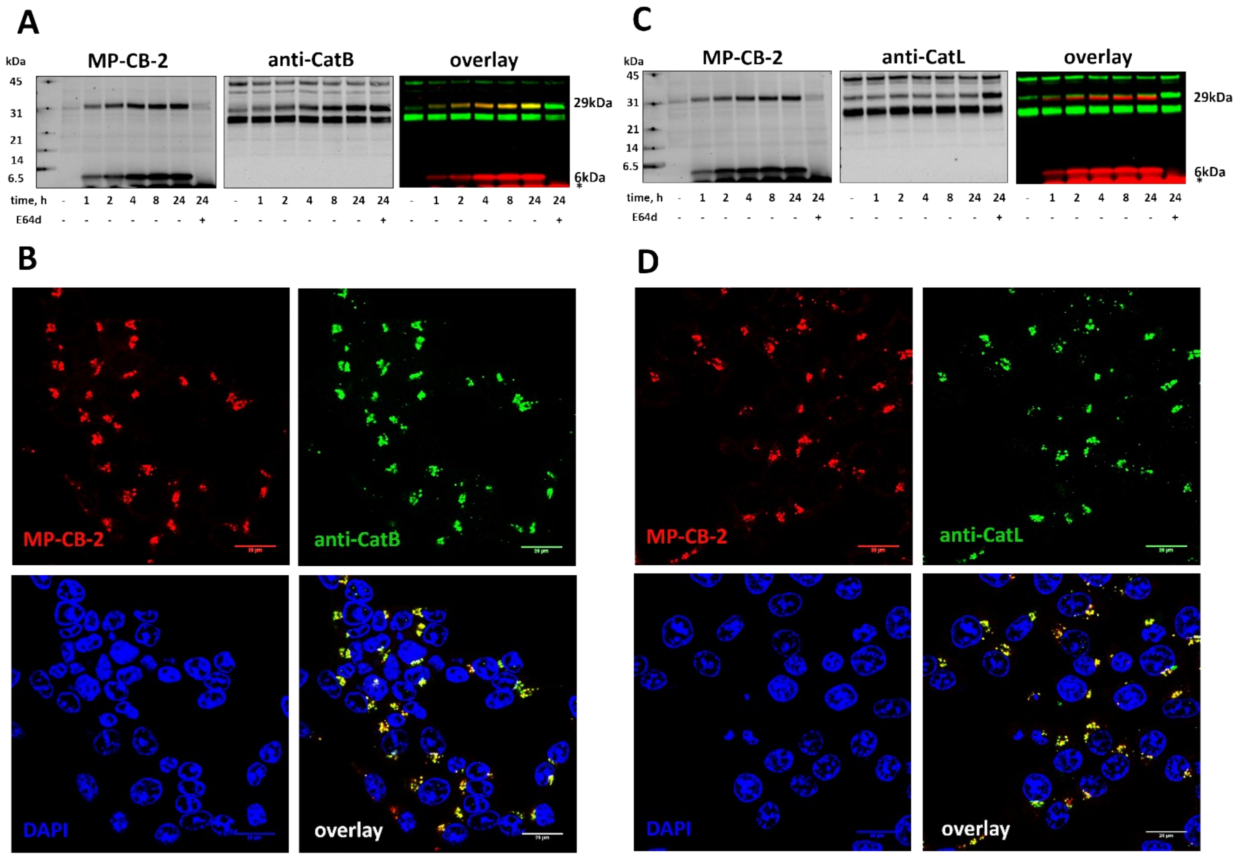

Figure 13 Cathepsin B labeling in HCT-116 cancer cells using MP-CB-2 probe. Panel A and C. Cathepsin B was selectively labeled in HCT-116 cell line using 1 μM MP-CB-2 ABP (Panel A). This probe labels single chain of cathepsin B form (~29 kDa) and also a light chain (~6 kDa) which is formed after sample boiling. The cathepsin B labeling can be prevent by using E-64d inhibitor. MP-CB-2 probe does not label cathepsin L even after prolonged incubation (24 hours, Panel C). Asterix (*) indicates unbound MP-CB-2 probe that migrated through the whole gel. Time point "-" indicates no probe was added. **Panel B and D.** Localization of cathepsin B in HCT-116 cells using 1 μM of MP-CB-2 ABP (overnight incubation) and anti-cathepsin B antibody. MP-CB-2 and cat-B antibody greatly overlap, which confirms the high selectivity of the probe. The signal from the probe only partially overlaps with anti-cathepsin L antibody, demonstrating that both enzymes share the same subcellular localization, however there are also some cathepsin B-rich lysosomes lacking cathepsin L

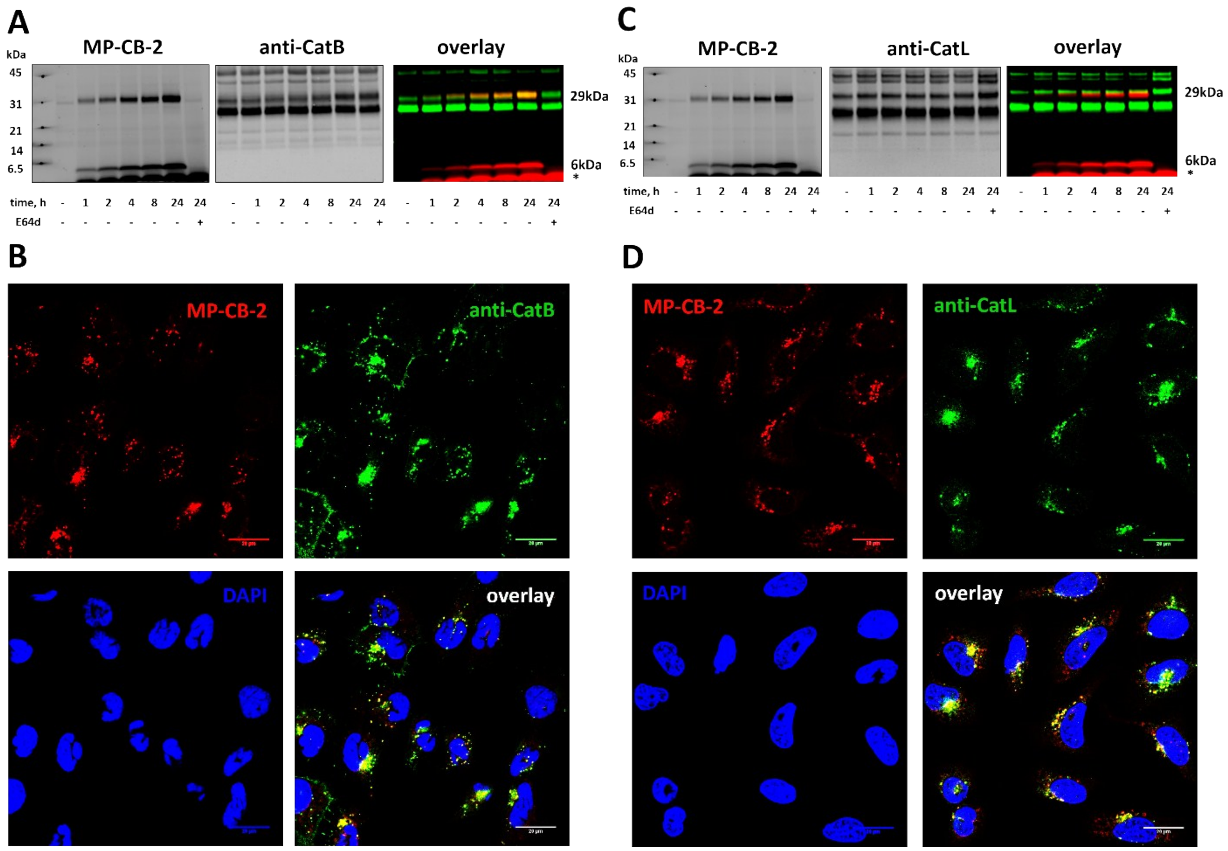

Figure 14 Cathepsin B labeling in U2-OS cancer cells using MP-CB-2 probe. Panel A and C. Cathepsin B was selectively labeled in U2-OS cell line using 1 μM MP-CB-2 ABP (Panel A). This probe labels single chain of cathepsin B form (~29 kDa) and also a light chain (~6 kDa) which is formed after sample boiling. The cathepsin B labeling can be prevent by using E-64d inhibitor. MP-CB-2 probe does not label cathepsin L even after prolonged incubation (24 hours, **Panel C**). Asterix (*) indicates unbound MP-CB-2 probe that migrated through the whole gel. Time point "-" indicates no probe was added. **Panel B and D.** Localization of cathepsin B in U2-OS cells using 1 μM of MP-CB-2 ABP (overnight incubation) and anti-cathepsin B antibody. MP-CB-2 and cat-B antibody greatly overlap, which confirms the high selectivity of the probe. The signal from the probe only partially overlaps with anti-cathepsin L antibody, demonstrating that both enzymes share the same subcellular localization, however there are also some cathepsin B-rich lysosomes lacking cathepsin L

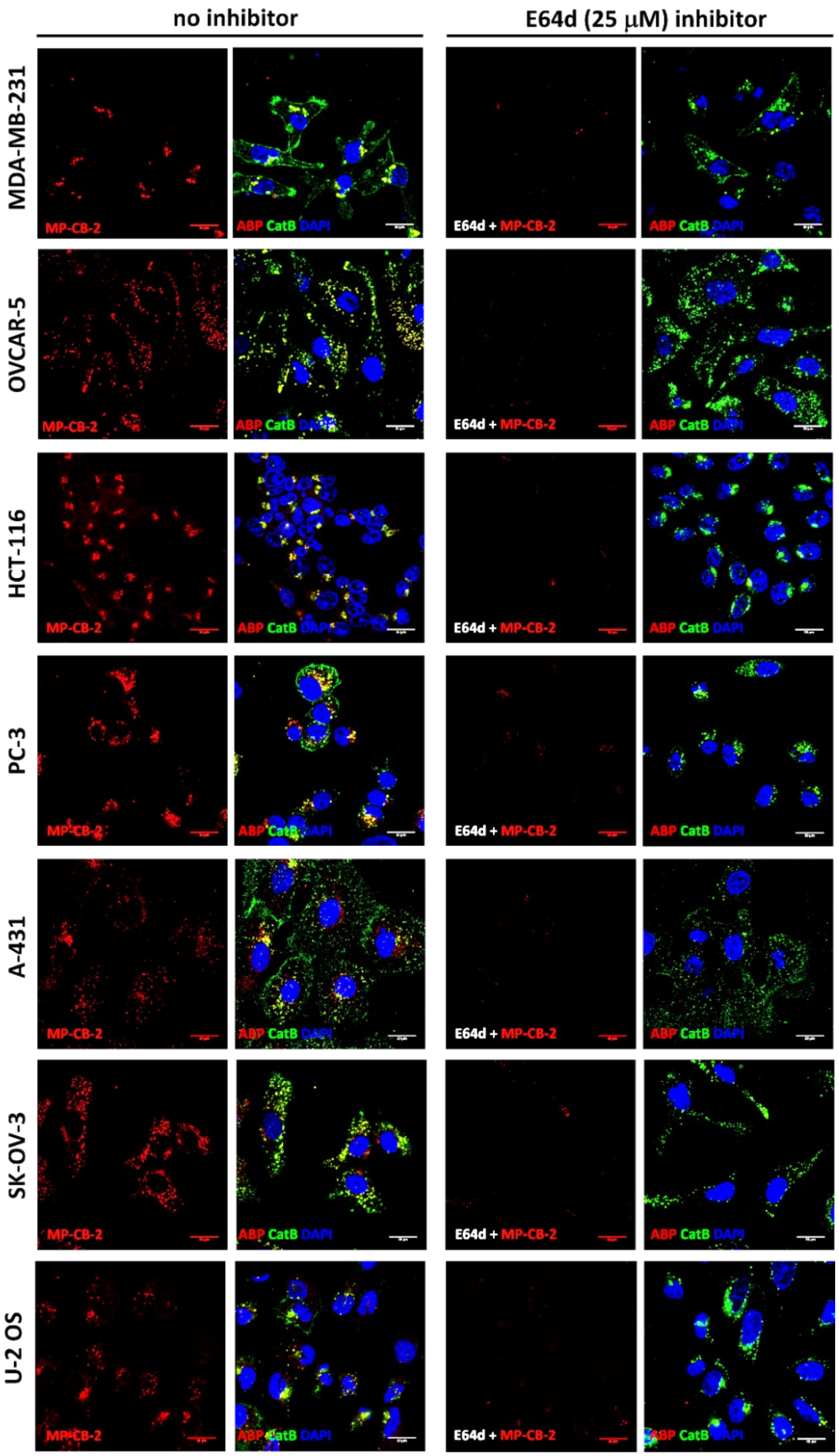

Figure 15 Cathepsin B labeling in the presence of E-64d inhibitor. Cells were preincubated with pan-cathepsins cell-permeable E-64d inhibitor (25 µM) for 4 hours (or left untreated), followed by cathepsin B labeling with MP-CB-2 probe (1 µM, overnight incubation). Next, cells were processed and subjected to immunofluorescence imaging as described in the Material and Methods section. Scale bar is 20 µm.

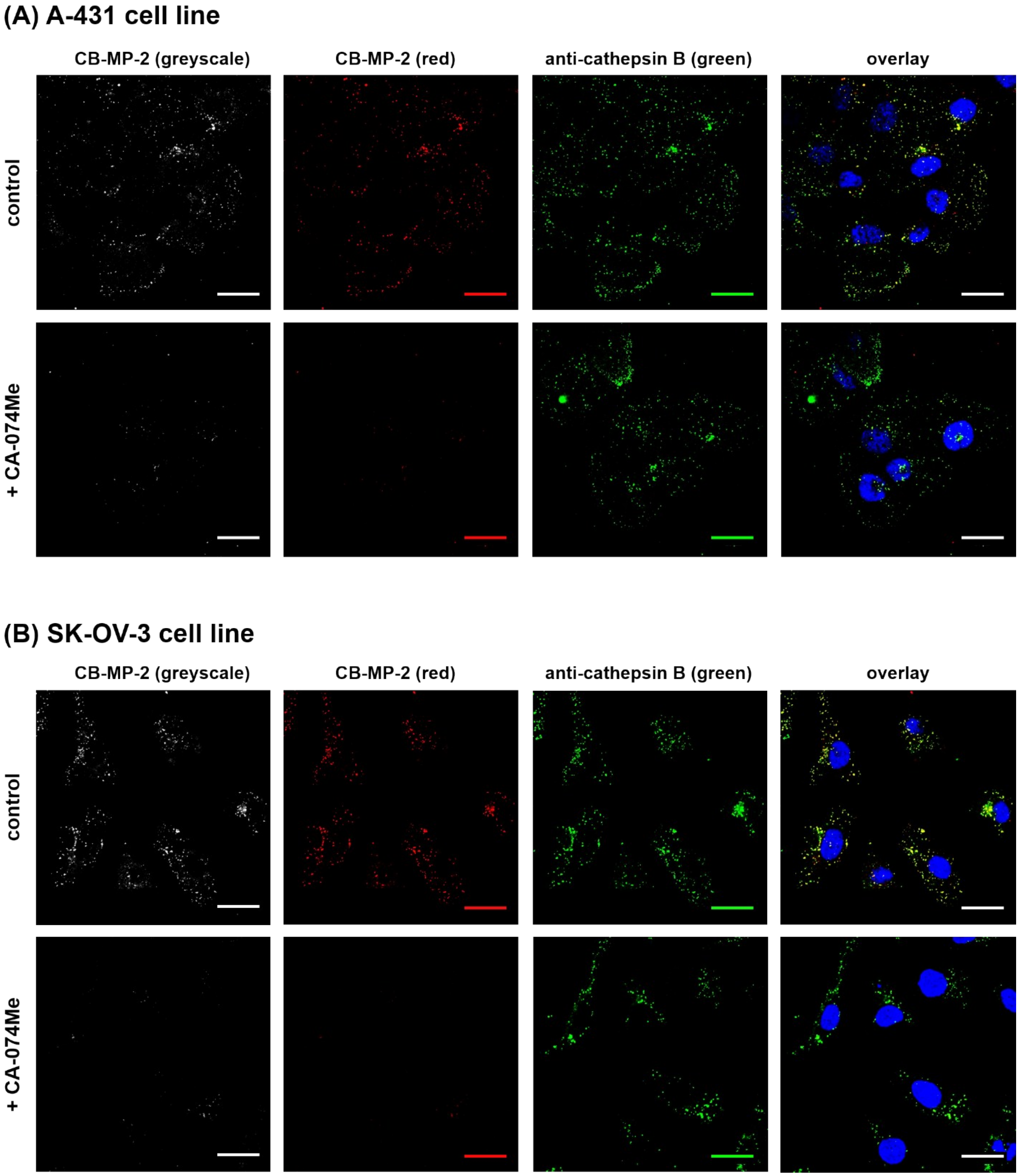

Figure 16 Cathepsin B labeling in the presence of cathepsin B selective CA-074Me inhibitor. Cathepsin B-poor cells (A431) and cathepsin B rich cells (SK-OV-3) were preincubated with cathepsin B cell-permeable CA-074Me inhibitor (25 μM) for 4 hours (or left untreated), followed by cathepsin B labeling with MP-CB-2 probe (1 μM, 12 hour incubation). Next, cells were processed and subjected to immunofluorescence imaging as described in the Material and Methods section. Scale bar is 25 μm.

picture no.	weighted correlation coefficient (wcc) **MP-CB-2 probe** and **cathespin B antibody**						
	A-431	U2-OS	PC-3	OVCAR-5	SK-OV-3	HCT-116	MDA-MB-231
**1**	0.959	0.966	0.934	0.901	0.948	0.971	0.905
**2**	0.921	0.99	0.884	0.905	0.932	0.991	0.945
**3**	0.955	0.967	0.875	0.928	0.946	0.983	0.921
**4**	0.93	0.965	0.915	0.947	0.952	0.995	0.898
**5**	0.922	0.94	0.915	0.924	0.951	0.993	0.923
**6**	0.888	0.981	0.909	0.917	0.948	0.98	0.956
**7**	0.925	0.985	0.941	0.96	0.939	0.972	0.966
**8**	0.961		0.957		0.93		
**Average**	**0.933**	**0.971**	**0.916**	**0.926**	**0.943**	**0.984**	**0.931**
**SD**	0.025	0.017	0.028	0.021	0.009	0.010	0.026

picture no.	weighted correlation coefficient (wcc) **MP-CB-2 probe** and **cathespin L antibody**						
	A-431	U2-OS	PC-3	OVCAR-5	SK-OV-3	HCT-116	MDA-MB-231
**1**	0.877	0.937	0.78	0.79	0.856	0.966	0.743
**2**	0.704	0.889	0.671	0.773	0.875	0.935	0.719
**3**	0.773	0.9	0.744	0.864	0.93	0.982	0.666
**4**	0.755	0.919	0.718	0.834	0.877	0.954	0.626
**5**	0.802	0.913	0.608	0.78	0.916	0.912	0.727
**6**	0.785	0.945	0.615	0.694	0.927	0.889	0.661
**7**	0.745	0.922	0.663	0.755	0.858	0.921	0.711
**8**			0.66	0.844			0.7
**Average**	**0.777**	**0.918**	**0.682**	**0.792**	**0.891**	**0.937**	**0.694**
**SD**	0.054	0.020	0.061	0.055	0.032	0.033	0.040

Table 4 Weighted correlation coefficient (wcc) between MP-CB-2 probe and cathepsin B (upper panel) and MP-CB-2 probe and cathepsin L (bottom panel) calculated based on at least seven whole pictures analysis. Weighted correlation coefficients were calculated using ZEN 2011 software.

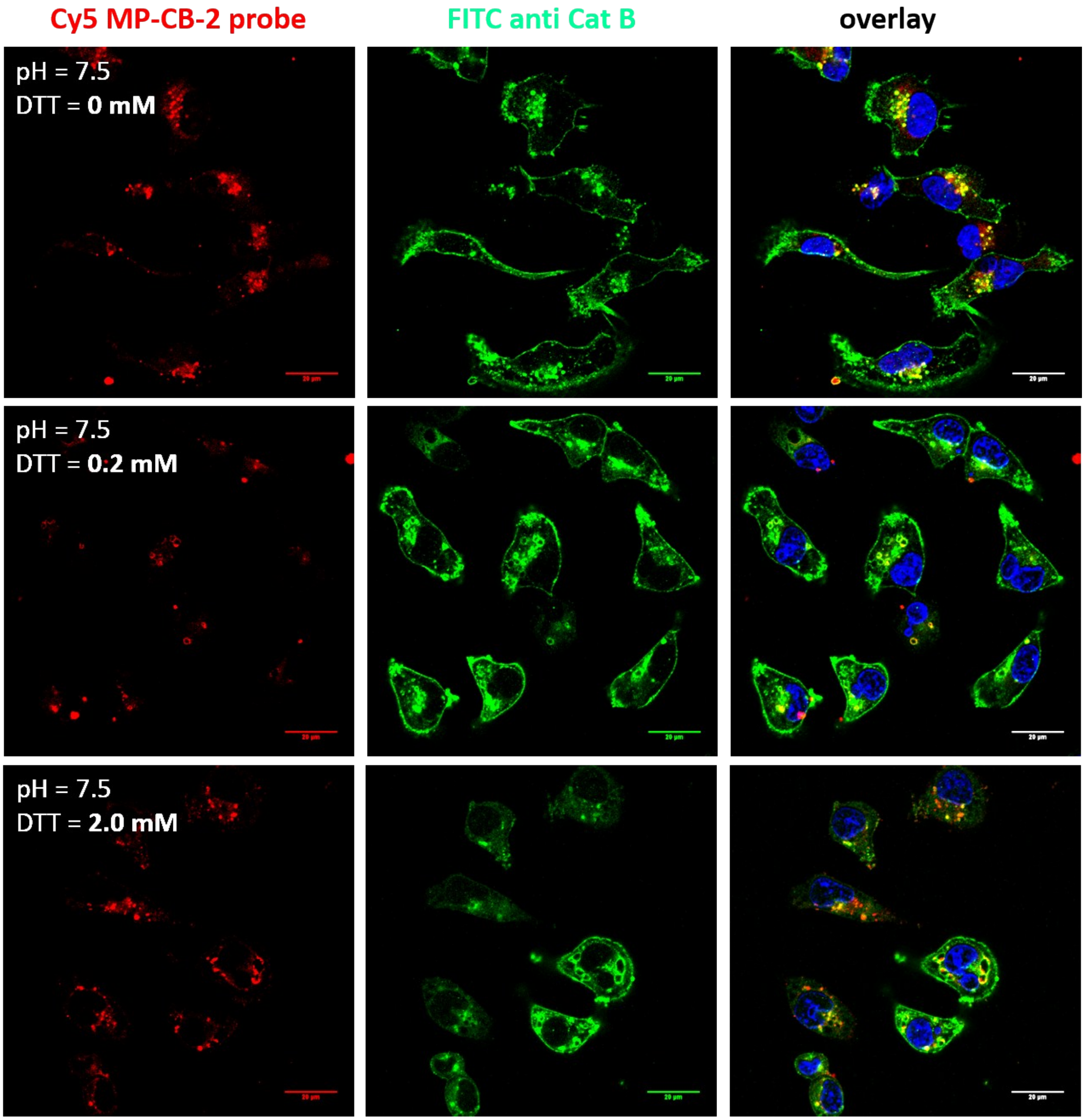

Figure 17 Cathepsin B labeling in MDA-MB-231 cells in pH 7.5 with various concentration of a reducing agent DTT (0-2.0 mM). MDA-MB-231 cells were supplemented with DTT, and immediately after incubated with 1 µM of MP-CB-2 probe overnight. Next, cells were processed and subjected to immunofluorescence imaging as described in the Material and Methods section. Scale bar is 20 µm.

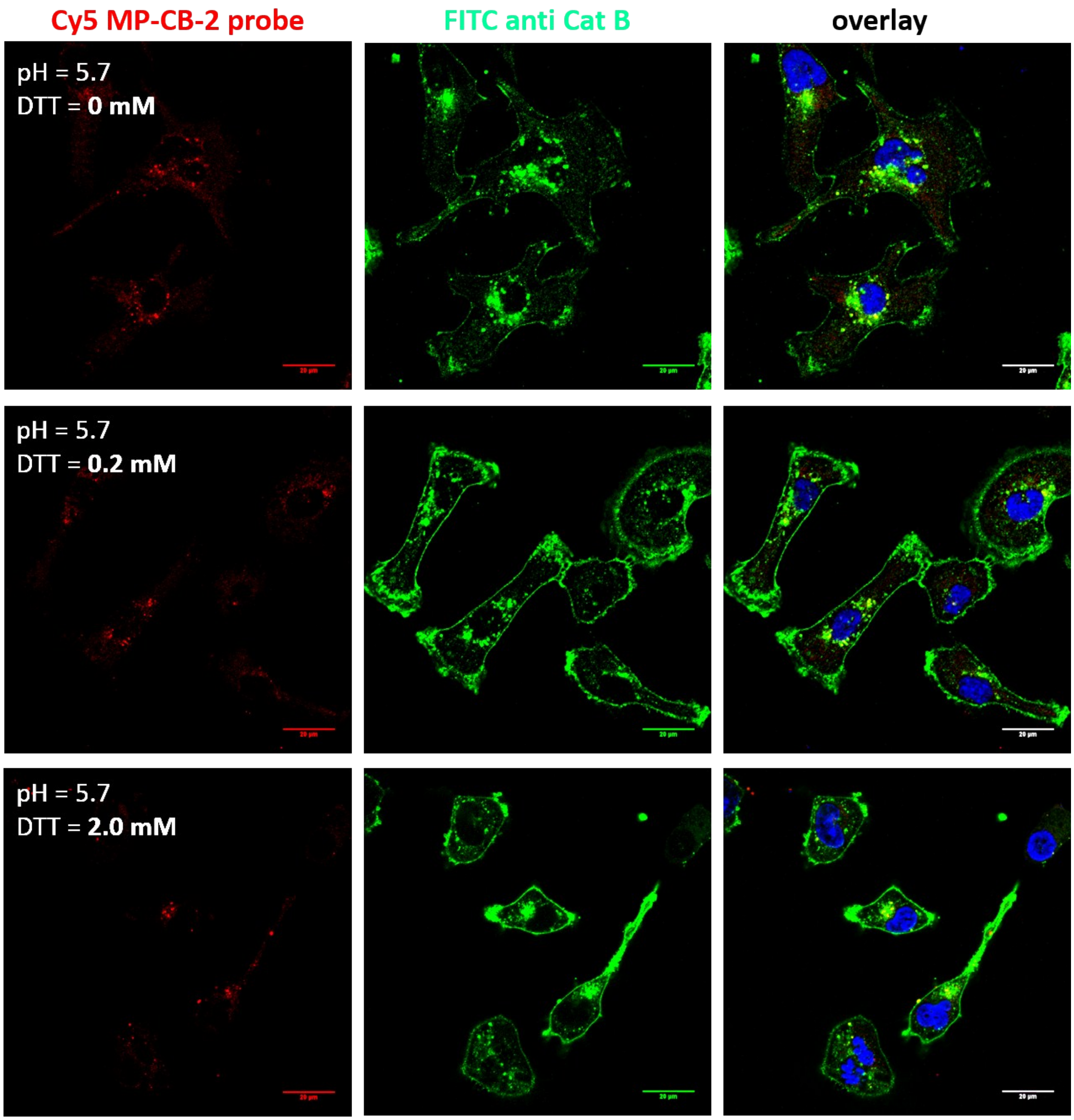

Figure 18 Cathepsin B labeling in MDA-MB-231 cells in pH 5.7 with various concentration of a reducing agent DTT (0-2.0 mM). MDA-MB-231 cells were supplemented with DTT, and immediately after incubated with 1 µM of MP-CB-2 probe overnight. Next, cells were processed and subjected to immunofluorescence imaging as described in the Material and Methods section. Scale bar is 20 µm.

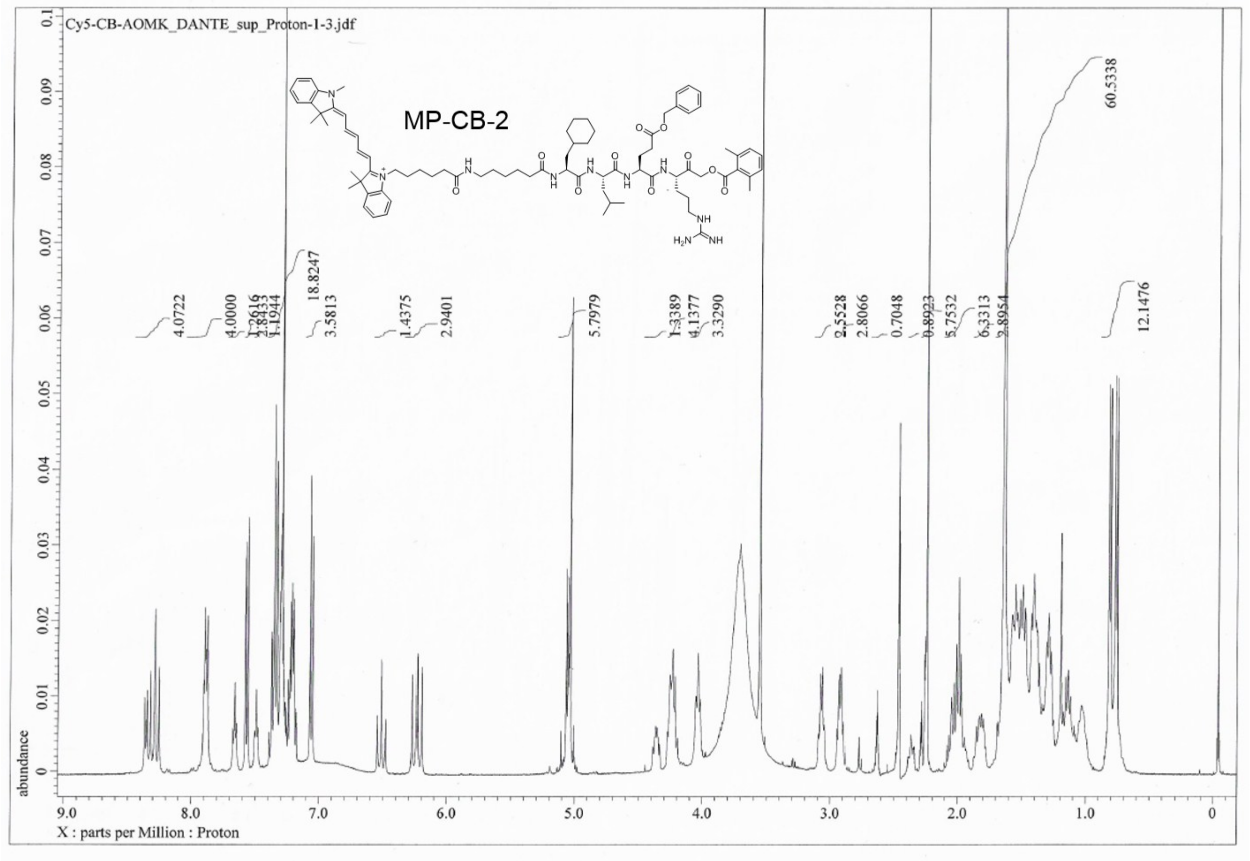

Figure 19 ^{1}H NMR spectrum of MP-CB-2 activity-based probe.

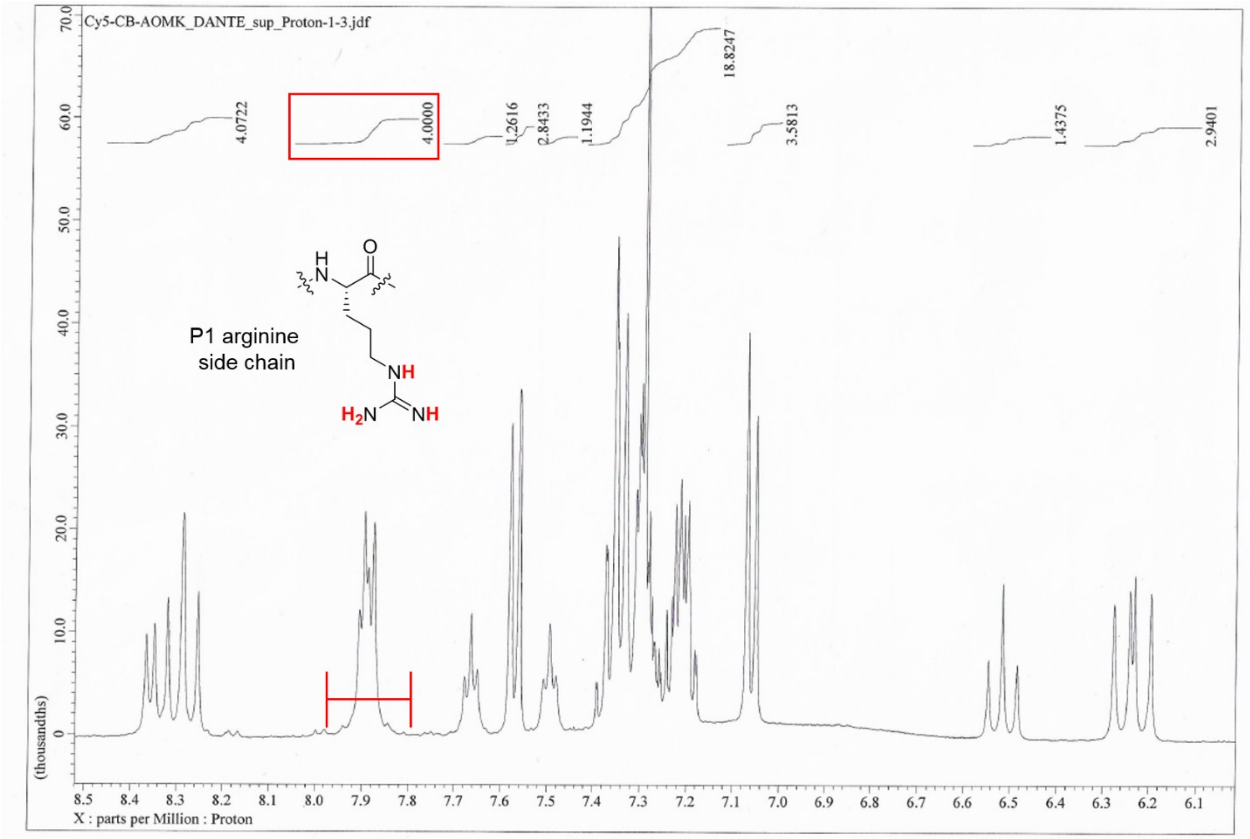

Figure 20 ^{1}H NMR spectrum of MP-CB-2 activity-based probe presented in the 6.0 – 8.5 ppm range. The integration of signal from protons located on a guanidine group (m, **4H**, 7.85-7.95) indicates that the Cy5-dye was attached to the free-N terminal of H_2N-ahx-Cha-Leu-Glu(*O*-Bzl)-Arg-AOMK compound, but not to the guanidine group (please see MP-CB-2 synthetic route for details).

Substrate	$[m/z + H]^+$ calculated	$[m/z + H]^+$ measured
Ac-Arg-Cha-hSer(Bzl)-Nle-ACC	888.4978	888.4988
Ac-Arg-Nle-Arg-Ser(Bzl)-ACC	439.2376 (z = +2)	439.2355 (z = +2)
Ac-Nva-Leu-Glu(Chx)-Ser(Bzl)-ACC	861.4393	861.4351
Ac-2Aoc-Cha-Orn-Ser(Bzl)-ACC	846.476	846.4711
Ac-hLeu-Nle-hSer(Bzl)-Arg-ACC	848.4665	848.4666
Ac-hCha-Leu-hSer(Bzl)-Arg-ACC	888.4978	888.4987
Ac-Phe(4Cl)-2Aoc-hSer(Bzl)-Arg-ACC	930.4276	930.4287
Ac-Nva-Cha-Glu(Bzl)-Arg-ACC	888.4614	888.4615
Ac-Phe(4Cl)-hCha-Glu(Chx)-Arg-ACC	976.4694	976.4655
Ac-Leu-2Aoc-hCha-Arg-ACC	838.5186	838.5122
Ac-Cha-Leu-Glu(Bzl)-Arg-ACC	902.4771	902.4787
Ac-Met-Arg-3Pal-Arg-ACC	426.7003 (z = +2)	426.7006 (z = +2)
Ac-Phe(4Cl)-Leu-hSer(Bzl)-Arg-ACC	902.3963	902.3955
Ac-Cha-Leu-hSer(Bzl)-Arg-ACC	874.4822	874.4851
Ac-hLeu-Nle-Glu(Bzl)-Arg-ACC	876.4614	876.4659
Ac-hTyr-Leu-Glu(Bzl)-Arg-ACC	926.4407	926.4455

Ac-hPhe-Leu-Glu(Bzl)-Arg-ACC	910.4458	910.4450
Ac-hLeu-Nle-Glu(Chx)-Arg-ACC	868.4927	868.4212

Table 5 HR-MS analysis of ACC-labeled tetrapeptide substrates selective for cathepsin B. All substrates were purified on semi-preparative HPLC to at least 95% of purity.

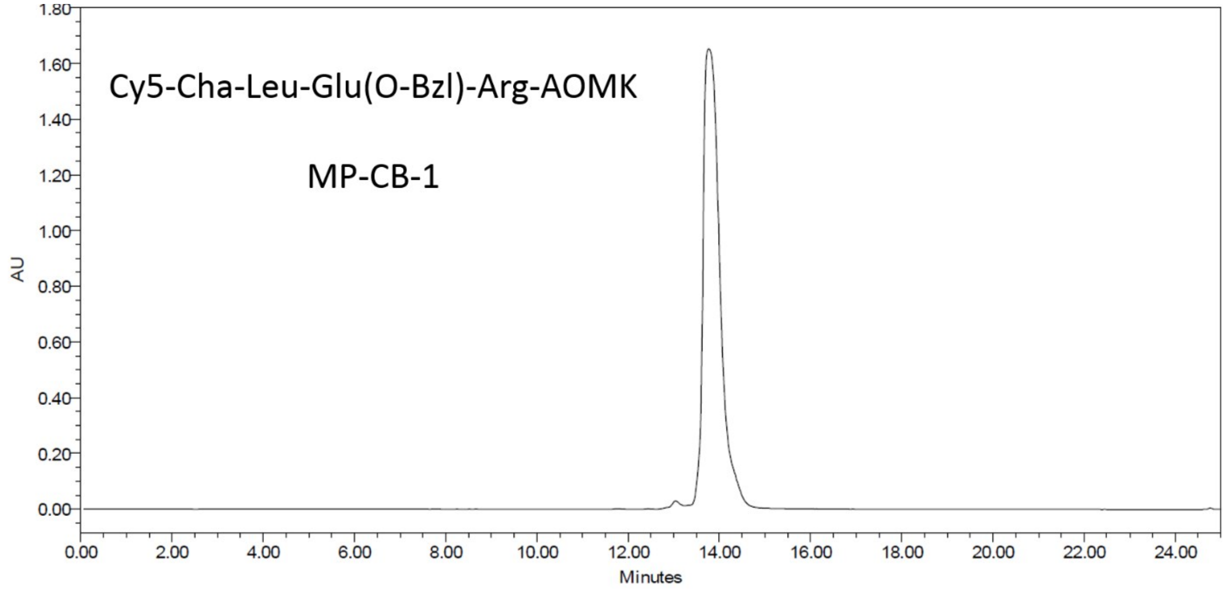
Cy5-Cha-Leu-Glu(O-Bzl)-Arg-AOMK
MP-CB-1
AU
Minutes

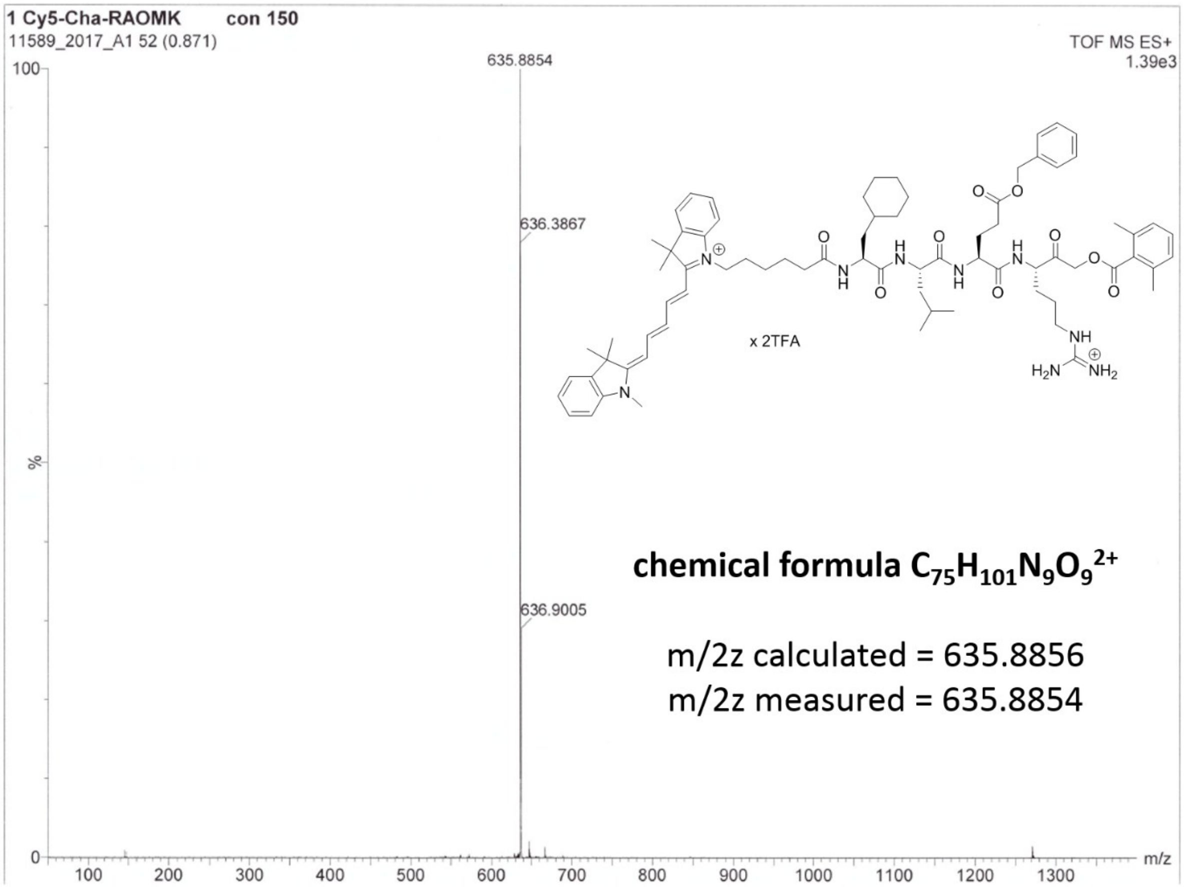
1 Cy5-Cha-RAOMK con 150
11589_2017_A1 52 (0.871)
TOF MS ES+
1.39e3
635.8854
636.3867
636.9005
x 2TFA
%
m/z
chemical formula $C_{75}H_{101}N_9O_9^{2+}$
m/2z calculated = 635.8856
m/2z measured = 635.8854

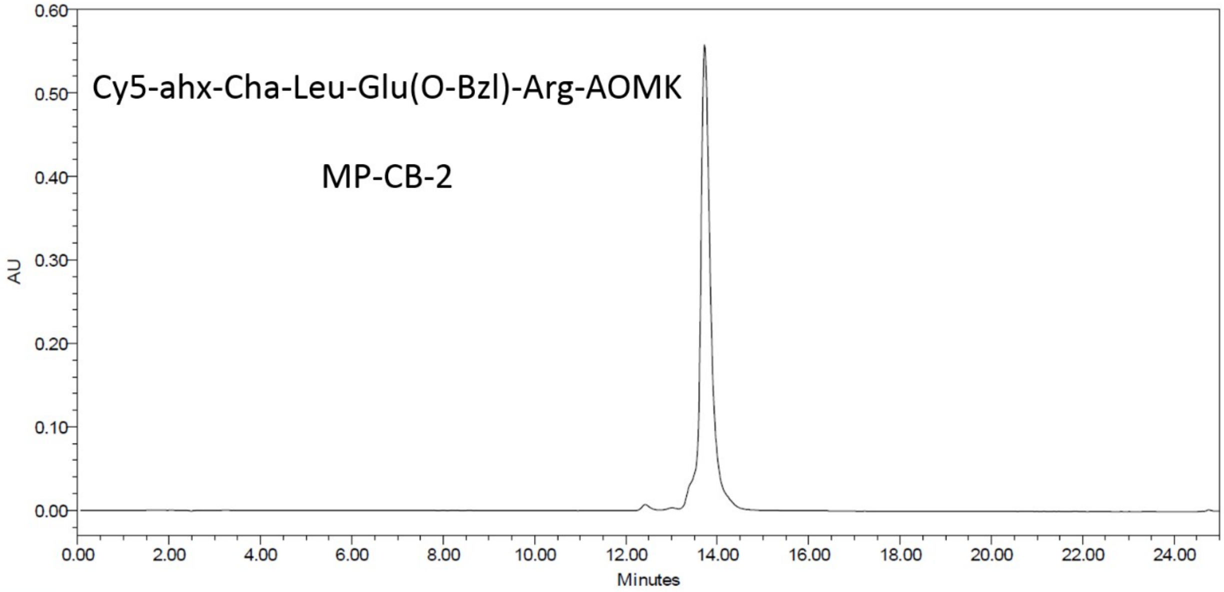

Cy5-ahx-Cha-Leu-Glu(O-Bzl)-Arg-AOMK
MP-CB-2
AU
0.60
0.50
0.40
0.30
0.20
0.10
0.00
0.00 2.00 4.00 6.00 8.00 10.00 12.00 14.00 16.00 18.00 20.00 22.00 24.00
Minutes

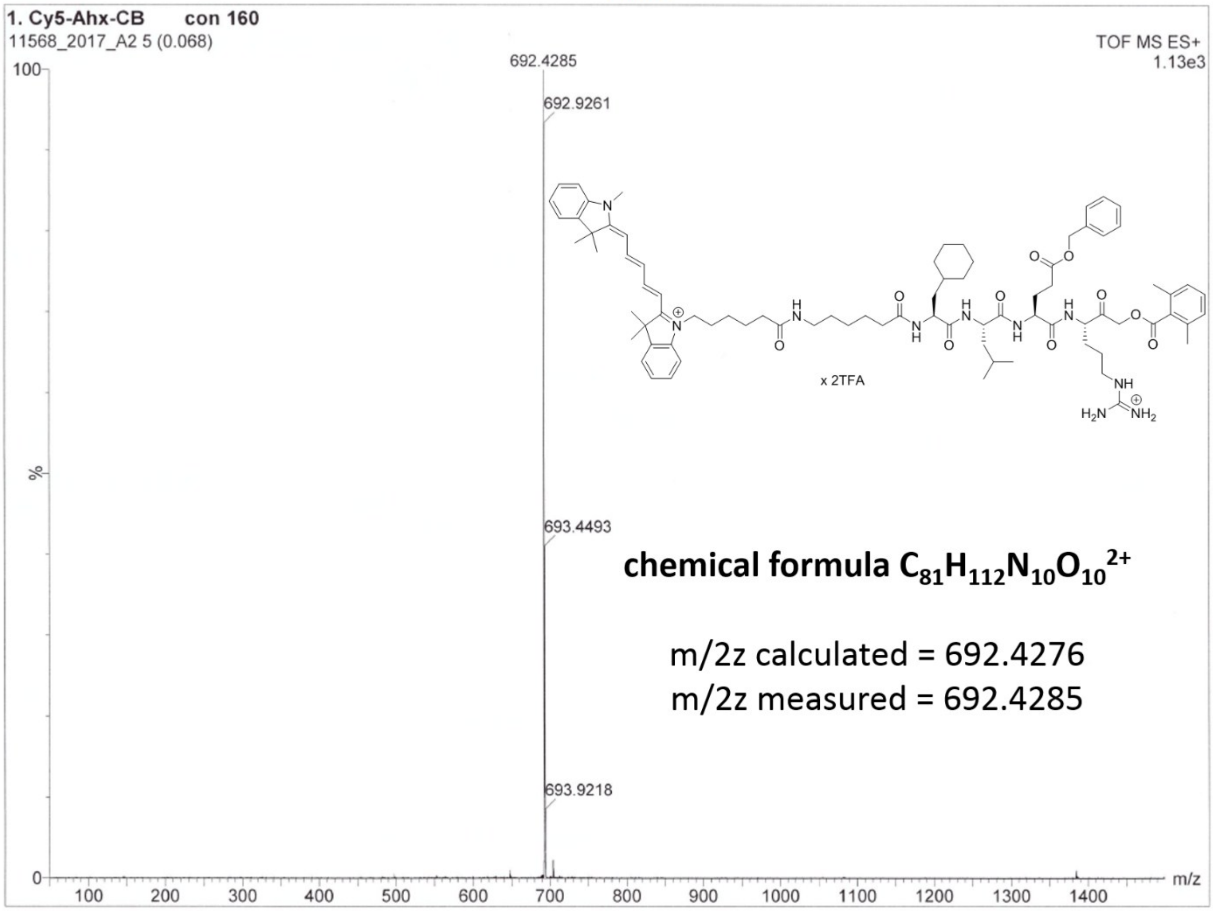

1. Cy5-Ahx-CB con 160
11568_2017_A2 5 (0.068)
TOF MS ES+
1.13e3
692.4285
692.9261
693.4493
693.9218
100
%
0
100 200 300 400 500 600 700 800 900 1000 1100 1200 1300 1400
m/z
x 2TFA
chemical formula $C_{81}H_{112}N_{10}O_{10}^{2+}$
m/2z calculated = 692.4276
m/2z measured = 692.4285

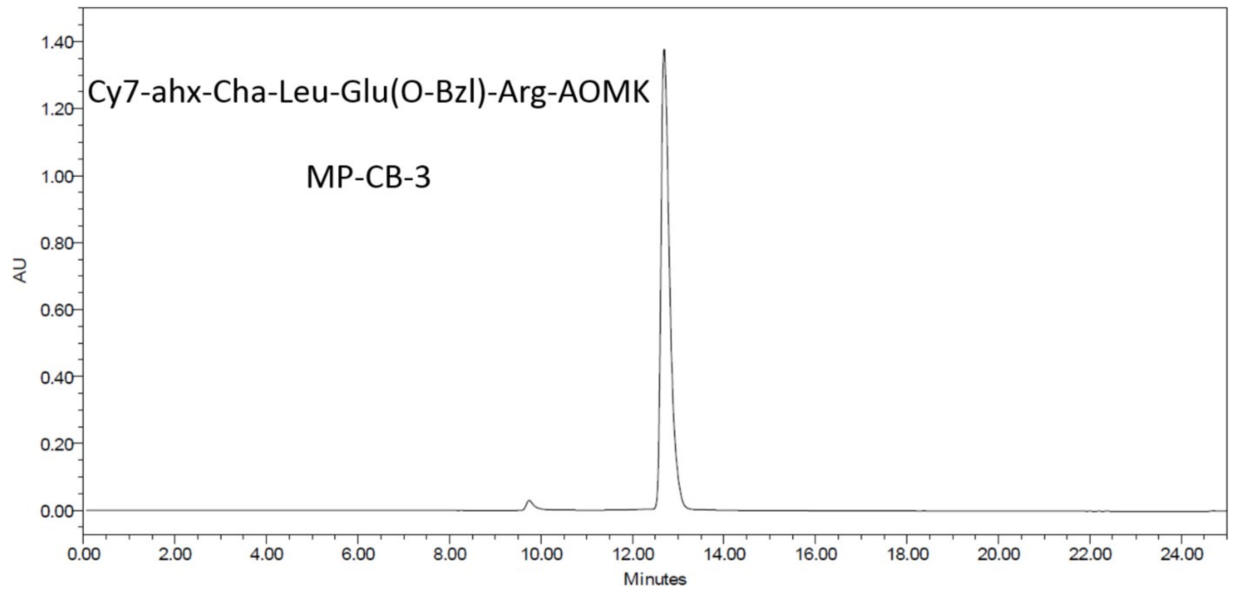
Cy7-ahx-Cha-Leu-Glu(O-Bzl)-Arg-AOMK
MP-CB-3
AU
1.40
1.20
1.00
0.80
0.60
0.40
0.20
0.00
0.00
2.00
4.00
6.00
8.00
10.00
12.00
14.00
16.00
18.00
20.00
22.00
24.00
Minutes

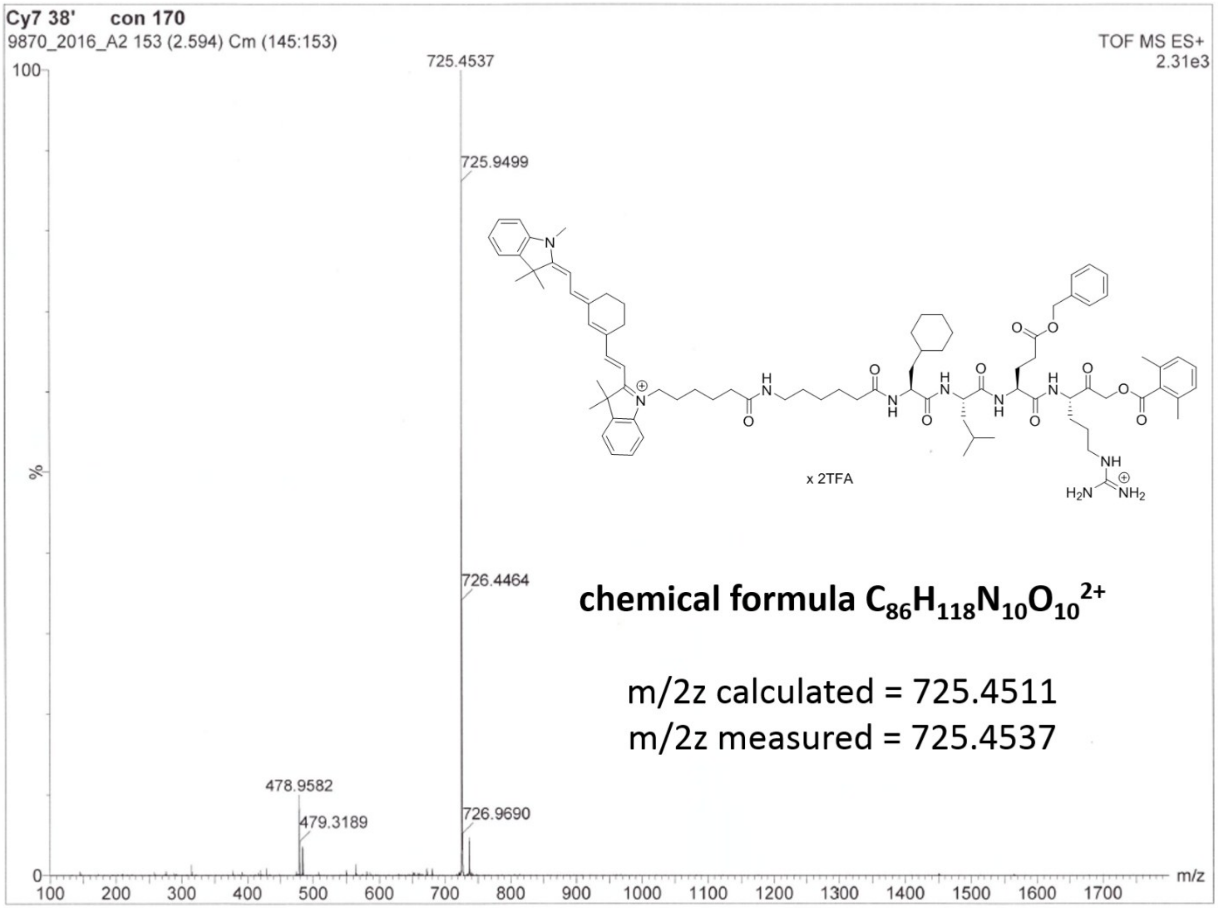
Cy7 38' con 170
9870_2016_A2 153 (2.594) Cm (145:153)
TOF MS ES+
2.31e3
725.4537
725.9499
726.4464
478.9582
479.3189
726.9690
x 2TFA
chemical formula $C_{86}H_{118}N_{10}O_{10}^{2+}$
m/2z calculated = 725.4511
m/2z measured = 725.4537
%
m/z
100
200
300
400
500
600
700
800
900
1000
1100
1200
1300
1400
1500
1600
1700
